# Astrocyte Senescence Disrupts the Extracellular Mitochondrial Compartment and Compromises Bioenergetic Support to Human Neurons

**DOI:** 10.3390/antiox15091127

**Published:** 2026-09-06

**Authors:** Pedro Amorim, Lívia de Sá Hayashide, Vitor Emanuel Leocadio, Mariana Marques, Isabelle Navarra, Cherley Borba Vieira Andrade, Jorge José de Carvalho, Rafael Serafim Pinto, Luan Pereira Diniz

**Affiliations:** 1 Laboratório de Investigação Metabólica Associada ao Envelhecimento, Instituto de Ciências Biomédicas, Universidade Federal do Rio de Janeiro, Rio de Janeiro 21941-902, Brazil; 2 Departamento de Histologia e Embriologia, Instituto de Biologia Roberto Alcântara Gomes, Universidade do Estado do Rio de Janeiro, Rio de Janeiro 20551-030, Brazil; 3 Instituto de Educação Médica (IDOMED), Campus Vista Carioca, Rio de Janeiro 20071-004, Brazil

**Keywords:** astrocyte senescence, oxidative stress, mitochondrial dysfunction, extracellular mitochondria, human astrocytes, astrocyte–neuron communication, neuronal bioenergetics, brain aging, doxorubicin

## Abstract

Astrocyte senescence is a recognized feature of brain aging, but its impact on neuronal mitochondrial homeostasis remains poorly defined, particularly in human cells. Here we show that doxorubicin-induced senescence disrupts mitochondrial function in primary human astrocytes and compromises their capacity to sustain neuronal bioenergetics. Senescent astrocytes accumulated a denser population of smaller, ultrastructurally damaged mitochondria together with increased levels of fission, fusion and biogenesis-associated proteins. Despite this apparent expansion of the mitochondrial compartment, these cells displayed reduced mitochondrial membrane potential, intracellular ATP and cellular metabolic activity, indicating accumulation of a functionally impaired mitochondrial population. Senescence also remodeled the extracellular mitochondrial compartment: conditioned medium from senescent astrocytes contained fewer mitochondrial particles with lower membrane potential and reduced ATP. Functionally, conditioned medium from control astrocytes increased TOMM20 and PGC-1α levels in human postmitotic neurons, whereas medium from senescent astrocytes failed to elicit this response and instead promoted hydrogen peroxide accumulation, ATP depletion and reduced cellular metabolic activity in the absence of overt cytotoxicity. Neurons acquired an astrocyte-derived MitoTracker signal from both conditions. Our data indicate that factors released by senescent human astrocytes are sufficient to induce neuronal mitochondrial and redox dysfunction.

## 1. Introduction

The human brain has exceptionally high energetic demands and depends on continuous interactions between neurons and glial cells to maintain tissue homeostasis [1]. Among glial populations, astrocytes regulate extracellular ion balance, neurotransmitter recycling, blood–brain barrier function, antioxidant defenses, and the supply of metabolic substrates to neurons [2]. They also actively modulate neuronal activity, synaptic function, and tissue responses to injury. Accordingly, astrocyte dysfunction has been increasingly associated with brain aging and neurodegenerative disorders [3].

Much of our current understanding of astrocyte biology is derived from rodent models. However, human astrocytes differ from their rodent counterparts in morphology, cellular complexity, gene expression, calcium signaling, and metabolic organization [4]. These differences highlight the importance of human cellular models for investigating mechanisms directly relevant to human brain aging and disease.

Cellular senescence is a stress-induced state characterized by persistent cell-cycle arrest, DNA damage signaling, chromatin and metabolic remodeling, and acquisition of a senescence-associated secretory phenotype [5]. Senescent astrocytes have been identified in the context of brain aging and Alzheimer’s disease and may disrupt neuronal homeostasis by reducing metabolic and trophic support, promoting chronic inflammation, and impairing glutamate clearance [6,7]. These changes may progressively compromise neuronal resilience and increase susceptibility to degeneration [8].

Experimental models of senescence in human astrocytes remain limited. Our group recently established a model of doxorubicin-induced senescence in primary human astrocytes [9]. Transient doxorubicin exposure induced a stable phenotype characterized by increased senescence-associated β-galactosidase activity, persistent activation of p21^Cip1^ and p53, sustained DNA damage signaling, and increased expression of senescence-associated secretory factors, including IL-6, IL-1β, and MMP3. These alterations persisted after drug withdrawal, supporting the use of this model to investigate the long-term functional consequences of human astrocyte senescence.

Mitochondrial dysfunction is a central feature of both cellular senescence and brain aging. Astrocytic mitochondria contribute to redox regulation, calcium signaling, metabolic flexibility, and neuronal support. Their functional integrity depends on coordinated mitochondrial biogenesis, fusion, fission, and mitophagy [10]. Disruption of these quality-control mechanisms can lead to the accumulation of fragmented, depolarized, or structurally damaged mitochondria, reduced ATP production, and increased oxidative stress [11].

Previous studies from our group showed that aging-associated stress impairs mitophagy, disrupts mitochondrial homeostasis, and increases the vulnerability of differentiated murine astrocytes to oxidative damage and cell death [12]. Consistent with these findings, aging has been associated with increased mitochondrial fragmentation and altered mitochondrial dynamics in astrocytes [13], while mitochondrial abnormalities and astrocytic atrophy have been reported in the aged human cortex [14].

Evidence from human cellular models further indicates that astrocyte mitochondrial competence directly influences neuronal function. Human induced pluripotent stem cell-derived astrocytes with mitochondrial complex I deficiency impair neuronal physiology, particularly under inflammatory conditions [15]. Conversely, metabolically competent astrocytes can support dopaminergic neurons carrying respiratory-chain defects [16]. Thus, astrocytes may either preserve or compromise neuronal homeostasis depending on their own metabolic and mitochondrial state.

In addition to soluble metabolites and trophic factors, astrocytes can communicate with neurons through the extracellular release and intercellular transfer of mitochondria. Astrocyte-derived mitochondria have been implicated in neuronal rescue and recovery following cerebral ischemia [17,18]. However, the biological effects of this communication are likely to depend on the quantity and functional quality of the released mitochondrial material. Mitochondria derived from healthy astrocytes may support neuronal bioenergetics, whereas mitochondrial particles released by dysfunctional or senescent astrocytes may provide insufficient support or contribute to cellular stress. Nevertheless, it remains unknown how senescence affects mitochondrial release by human astrocytes and whether senescent astrocytes directly alter neuronal mitochondrial function.

In the present study, we used primary human astrocytes and human postmitotic neurons to investigate whether astrocyte senescence propagates mitochondrial damage across neural cell populations. We show that senescent human astrocytes accumulate structurally damaged and functionally impaired mitochondria, release fewer and less polarized extracellular mitochondrial particles, and lose their capacity to sustain neuronal mitochondrial homeostasis. Conditioned medium from control astrocytes enhanced neuronal mitochondrial content and biogenesis-associated signaling, whereas conditioned medium from senescent astrocytes induced oxidative stress, ATP depletion, and reduced cellular metabolic activity. These findings support a model in which senescent astrocytes act as an upstream source of mitochondrial dysfunction, propagating bioenergetic damage to human neurons through altered paracrine communication.

## 2. Materials and Methods

### 2.1. Primary Human Astrocyte Cultures

Commercially available human cortical astrocytes (Thermo Fisher, Waltham, MA, USA, cat. K1884) were used in this study. The cells were cultured in DMEM/F12 medium supplemented with 10% FBS on substrates pre-coated with poly-L-lysine. The cells were maintained in vitro at 37 °C in a humidified incubator with 5% CO_2_ and 95% air until they reached confluence. The culture medium was replaced every 2–3 days. At passage 1, the cells were expanded and cryopreserved. Cells were used for experiments up to passage 7. Routine morphological characterization of the cultures was performed, including immunostaining for astrocyte markers such as GFAP, glutamine synthetase, GLT1, S100 and aquaporin 4, to confirm the astrocytic identity and purity of the cultures. Cultures were routinely screened for mycoplasma contamination by morphological inspection of nuclear DAPI staining at 100× magnification; no evidence of contamination was observed throughout the experimental period. All experiments were performed using human astrocytes derived from the same commercial donor. Each *n* represents an independent experiment conducted using a separate cell passage and independently established control and senescent cultures, rather than technical replicates or different wells.

### 2.2. Doxorubicin-Induced Senescent Phenotype

Confluent primary human astrocyte cultures were divided into two experimental groups: control (treated with PBS) and doxorubicin-treated. In the latter group, cells were exposed to 250 nM doxorubicin (Libbs Farmacêutica, Embu das Artes, SP, Brazil) for 72 h in serum-containing medium. Following this treatment period, the drug was removed, fresh complete medium was added, and the cultures were maintained for an additional 4 days in vitro to allow for the establishment of a stable senescent phenotype. The culture medium was replaced every two days, and 24 h prior to sample collection, cells were transferred to serum-free medium to reduce confounding effects of serum-derived factors. Senescence induction with this protocol has been characterized in detail previously [9].

### 2.3. Immunocytochemistry

Human astrocyte cultures were fixed with 4% paraformaldehyde in phosphate-buffered saline (PBS), pH 7.4, for 15 min at room temperature. After fixation, the cells were washed with PBS and incubated for 1 h at room temperature in a blocking and permeabilization solution containing 3% bovine serum albumin, 5% normal goat serum and 0.2% Triton X-100 in PBS.

The cultures were subsequently incubated overnight at 4 °C with primary antibodies against TOMM20 (rabbit anti-TOMM20, 1:1000; Abcam, Cambridge, UK, cat. ab186735), mitofusin 1 and mitofusin 2 (mouse anti-MFN1+2, 1:300; Abcam, cat. ab57602), phosphorylated dynamin-related protein 1 at serine 616 (rabbit anti-phospho-DRP1 Ser616, 1:100; Thermo Fisher Scientific, Waltham, MA, USA, cat. PA5-64821), PGC-1α (rabbit anti-PGC-1α, 1:1000; Thermo Fisher Scientific, cat. PA5-72948), VDAC1 (rabbit anti-VDAC1, 1:300; Thermo Fisher Scientific, cat. PA1-954A) and HSP60 (mouse anti-HSP60/HSPD1, 1:200; Developmental Studies Hybridoma Bank, Lowa City, IA, USA, RRID: AB_2617272).

Following primary antibody incubation, the cells were thoroughly washed with PBS and incubated with the appropriate Alexa Fluor 488- or Alexa Fluor 555-conjugated goat anti-rabbit or anti-mouse IgG secondary antibodies (Thermo Fisher Scientific) for 2 h at room temperature. Alexa Fluor 555-conjugated antibodies were used at 1:1000, whereas Alexa Fluor 488-conjugated antibodies were used at 1:300. Cell nuclei were counterstained with Hoechst 33342 (Thermo Fisher Scientific). For negative controls, primary antibodies were omitted and samples were incubated with secondary antibodies alone; no specific staining was detected. Antibody specificity was supported by manufacturer validation, previous use in the literature, and the expected subcellular staining patterns. Fluorescence images were acquired using a Leica SPE confocal microscope(Wetzlar, Germany), a Nikon TE2000 fluorescence microscope (Nikon Instruments Inc., Tokyo, Japan), or a Nexcope NIB-620FL fluorescence microscope (Ningbo Yongxin Optics Co., Ningbo, China). Acquisition settings were kept constant across experimental groups within each experiment.

### 2.4. Immunofluorescence Quantification

Fluorescence images were acquired using 40× or 60× objectives, depending on the assay. Multiple microscopic fields were analyzed from each independent culture, encompassing approximately 140–180 astrocytes or approximately 200 neurons per experimental condition. Measurements obtained from individual fields were averaged to generate a single value for each independent culture. Thus, the reported *n* represents independent cultures rather than individual cells or microscopic fields. Densitometry of immunocytochemistry images was performed using integrated density values generated with Fiji/ImageJ software (ImageJ version 1.54r; National Institutes of Health, Bethesda, MD, USA) Data were collected from at least 10 fields per coverslip. The integrated density value was divided by the number of cells in each field. Mitochondrial morphology was analyzed in ImageJ using a previously described mitochondrial morphology macro [19,20]. Mitochondria were labeled with antibody anti-TOMM20. Mitochondrial structures within each cellular ROI were then segmented and quantified using the “Analyze Particles” function, allowing all morphometric parameters to be assessed on a per-cell basis. The number of mitochondrial particles was used as an estimate of mitochondrial density per cell, while the mean particle area was calculated as an indicator of average mitochondrial size. These morphometric parameters have been previously validated using well-characterized modulators of mitochondrial fission and fusion [12,13].

### 2.5. Measurement of Extracellular Hydrogen Peroxide

Extracellular H_2_O_2_ levels were quantified using the Amplex Red Hydrogen Peroxide/Peroxidase Assay Kit (Invitrogen, Thermo Fisher Scientific, cat. no. A22188). Following the manufacturer’s instructions with minor modifications. After treatment, 50 µL of culture supernatant was collected and incubated with 50 µL of reaction buffer containing 0.1 mM Amplex Red reagent and 0.2 U/mL horseradish peroxidase in 1× reaction buffer. The mixture was incubated for 30 min at room temperature in the dark. Fluorescence was measured using a GloMax microplate reader (Promega) with excitation at 520 nm and emission at 580–640 nm. Results were normalized to cell number and expressed as a percentage of the control.

### 2.6. Extracellular Lactate Levels

Extracellular lactate levels in the culture medium were assessed using a commercial kit (Labtest Diagnóstica S/A, Lagoa Santa, MG, Brazil, cat. 138). A total of 5 µL of medium was used for 50 µL of the reaction mix.

### 2.7. Nitrite Levels

Briefly, 50 µL of conditioned medium was mixed with 50 µL of 1% sulfanilamide, prepared in 10% phosphoric acid and incubated for 5 min at room temperature. Then, 50 µL of 0.1% *N*-(1-naphthyl)ethylenediamine prepared in water was added, followed by an additional 5 min incubation. Absorbance was measured at 540 nm.

### 2.8. Intracellular ATP Levels

Intracellular ATP levels were quantified using the CellTiter-Glo 2.0 luminescent assay (Promega, cat. G7572), according to the manufacturer’s instructions. Human astrocytes were cultured in 96-well plates, whereas neurons were cultured in 24-well plates. At the end of the experimental treatments, the culture medium was removed and 60 µL of CellTiter-Glo 2.0 reagent was added to each well of the 96-well plates containing astrocytes. The plates were incubated for 10 min at room temperature, after which 50 µL of the resulting lysate was transferred to a white opaque 96-well plate for luminescence measurement.

For neuronal cultures, 180 µL of CellTiter-Glo 2.0 reagent was added to each well of the 24-well plates. After a 10 min incubation at room temperature, the wells were scraped to ensure complete recovery of the cellular extract. Aliquots of 50 µL from each lysate were transferred to multiple wells of a white opaque 96-well plate, and the mean luminescence value of the technical replicates was calculated for each independent culture. Luminescence was measured using a GloMax plate reader (Promega), and ATP-dependent signals were normalized to the total protein content of the corresponding cellular extract.

### 2.9. Assessment of Mitochondrial Membrane Potential Using JC-1

Mitochondrial membrane potential was assessed using the JC-1 dye (Thermo Fisher Scientific, cat. T3168). Human astrocytes were seeded in 96-well plates at a density of 20,000 cells per well. After treatment, JC-1 was added to each well at a final concentration of 2 µg/mL, and the plate was incubated for 30 min at 37 °C in the dark. Following incubation, cells were washed twice with Gey’s balanced salt solution and maintained in phenol red-free DMEM/F12 during fluorescence acquisition. Readings were performed using a GloMax Discover plate reader (Promega). Red fluorescence (J-aggregates, indicative of polarized mitochondria) was measured at Ex/Em 520/580–640 nm, and green fluorescence (monomeric form, indicative of depolarized mitochondria) at Ex/Em 475/500–550 nm. Mitochondrial membrane potential was expressed as the red/green fluorescence ratio, normalized to the untreated control.

### 2.10. Cellular Metabolic Activity (MTT Assay)

Cellular metabolic activity was assessed using the MTT assay (3-[4,5-dimethylthiazol-2-yl]-2,5-diphenyltetrazolium bromide; Sigma-Aldrich, St. Louis, MO, USA, cat. M5655). Human astrocytes were seeded in 96-well plates at a density of 20,000 cells per well in 100 µL of culture medium. At the end of the experimental treatments, 10 µL of MTT solution (5 mg/mL in PBS) was added to each well, and the plates were incubated for 2 h at 37 °C in the dark. The culture medium was then carefully removed, and 100 µL of dimethyl sulfoxide (DMSO) was added to each well to dissolve the formazan crystals. The plates were agitated for 5 min at room temperature to ensure complete solubilization.

Neurons were cultured in 24-well plates in a final volume of 300 µL per well. After the experimental treatments, 30 µL of MTT solution (5 mg/mL in PBS) was added to each well, and the plates were incubated for 2 h at 37 °C in the dark. The culture medium was subsequently removed, and DMSO was added to dissolve the formazan crystals. The resulting solution from each neuronal culture was distributed into three wells of a 96-well plate, and the mean absorbance value of the technical replicates was calculated for each independent culture. Absorbance was measured at 560 nm using a GloMax microplate reader (Promega). Because MTT reduction depends on both mitochondrial and cytosolic dehydrogenase activity, results are reported as cellular metabolic activity rather than as an independent measure of cell viability. Values were expressed as a percentage relative to the untreated control group. Experiments were performed using at least three independent cultures.

### 2.11. Transmission Electron Microscopy and Mitochondrial Ultrastructural Analysis

Human astrocyte cultures were processed for qualitative and quantitative ultrastructural analysis by transmission electron microscopy, as previously described [12]. Cells were washed with PBS and detached from the culture plates by trypsinization. Trypsin activity was neutralized by adding complete culture medium, and the resulting cell suspension was collected and centrifuged at 350× *g* for 10 min. The supernatant was discarded, and the cell pellet was gently resuspended in 2.5% glutaraldehyde prepared in 0.1 M sodium cacodylate buffer, pH 7.2. Samples were maintained in the fixative for 24 h. After fixation, the samples were washed three times for 10 min each with 0.1 M sodium cacodylate buffer, pH 7.2. Post-fixation was performed with 1% osmium tetroxide in 0.1 M sodium cacodylate buffer, pH 7.2. The samples were then dehydrated through a graded acetone series (30%, 50%, 70%, 90% and 100%) and embedded in Poly/Bed 812 epoxy resin (Ted Pella Inc., Redding, CA, USA).

Following resin polymerization, ultrathin sections of approximately 70 nm were obtained using an ultramicrotome (Leica Microsystems, Wetzlar, Germany) and collected on 300-mesh copper grids. The sections were counterstained with 5% uranyl acetate and lead citrate and examined using a JEOL JEM-1011 transmission electron microscope (JEOL Ltd., Akishima, Tokyo, Japan). Digital electron micrographs were acquired using an ORIUS CCD digital camera (Gatan Inc., Pleasanton, CA, USA) at magnifications of 10,000× and 50,000×. At least five independent human astrocyte cultures were analyzed, with a minimum of three cells evaluated per independent culture. Quantitative analysis was performed using ImageJ. The number of damaged mitochondria was quantified in each microscopic field, and measurements obtained from the analyzed fields were averaged to generate a single value for each independent astrocyte culture. Mitochondria were classified as damaged based on previously established ultrastructural criteria, including disruption or discontinuity of the outer and inner mitochondrial membranes, abnormal or swollen mitochondrial morphology, loss or disorganization of mitochondrial cristae, and alterations in mitochondrial matrix electron density [12].

### 2.12. Preparation of Astrocyte-Conditioned Medium

Control and senescent human astrocytes were cultured in 6-well plates. At the end of the experimental treatments, the cultures were gently washed and maintained for 24 h in phenol red-free DMEM/F-12 medium (Gibco, Thermo Fisher Scientific, cat. 21041025). After 24 h, the astrocyte-conditioned medium was collected and centrifuged at 1500× *g* for 10 min to remove cells and cellular debris. This centrifugation speed was selected because it clears cells and large debris while leaving free mitochondria and mitochondria-containing particles in suspension, which typically require substantially higher centrifugal forces to sediment. The resulting supernatant was maintained on ice and used immediately either for neuronal treatment or for biochemical measurements. Neurons were maintained in the conditioned medium for 24 h and subsequently processed for biochemical or morphological analyses.

To generate mitochondria-depleted astrocyte-conditioned medium (mdCM), the clarified conditioned medium was passed through a sterile 0.22 µm syringe filter. This filtration step was used to remove extracellular mitochondrial particles from the conditioned medium, as previously described in studies investigating astrocyte-to-neuron mitochondrial transfer [17]. Astrocyte-conditioned medium was collected and clarified as described above to remove cells and cellular debris. An aliquot of the clarified conditioned medium was retained as the unfractionated conditioned medium. The remaining sample was centrifuged at 20,000× *g* for 30 min to sediment extracellular mitochondria and other large mitochondria-associated particulate material. The resulting supernatant was carefully collected without disturbing the pellet. The pellet was gently resuspended in phenol red-free DMEM/F12 and analyzed in parallel with the corresponding supernatant and unfractionated conditioned medium. ATP content in each fraction was quantified using the CellTiter-Glo luminescence assay (Promega) under equivalent experimental conditions.

### 2.13. Flow Cytometric Quantification of Extracellular Mitochondrial Particles

Astrocyte-conditioned medium was collected and incubated with 200 nM MitoTracker Green FM (Thermo Fisher Scientific, cat. M7514) for 30 min at 37 °C. After incubation, samples were immediately analyzed using a FACSCanto II flow cytometry system (BD Biosciences, San Jose, CA, USA). Unstained conditioned medium was used to establish background fluorescence, define the acquisition settings, and determine the threshold for MitoTracker Green-positive events. Cell-free medium incubated with MitoTracker Green under identical conditions was used to exclude probe-derived background events. Data were analyzed using FlowJo™ v10 software (FlowJo LLC, Ashland, OR, USA). Extracellular mitochondrial particles were identified as MitoTracker Green-positive events, and the number of mitochondrial particles detected in each sample was quantified. Values were normalized to those obtained from control astrocyte-conditioned medium.

### 2.14. Assessment of Extracellular Mitochondrial Membrane Potential

Astrocyte-conditioned medium was collected, and JC-1 was added to each sample at a final concentration of 2 µg/mL. Samples were incubated for 30 min at 37 °C in the dark. After incubation, aliquots were transferred to a black 96-well plate, and fluorescence was measured using a GloMax Discover microplate reader (Promega). Red fluorescence, corresponding to JC-1 aggregates and indicative of polarized mitochondria, was measured at Ex/Em 520/580–640 nm. Green fluorescence, corresponding to the monomeric form of JC-1 and indicative of mitochondrial depolarization, was measured at Ex/Em 475/500–550 nm. Extracellular mitochondrial membrane potential was expressed as the red-to-green fluorescence ratio, normalized to control astrocyte-conditioned medium.

### 2.15. Extracellular ATP Quantification

Extracellular ATP levels were quantified in astrocyte-conditioned medium using the CellTiter-Glo luminescence assay (Promega). Briefly, 50 µL of conditioned medium was transferred to each well of an opaque white 96-well plate, and an equal volume of CellTiter-Glo reagent was added. The plate was incubated for 30 min at room temperature to allow signal stabilization, and luminescence was measured using a GloMax microplate reader (Promega). Extracellular ATP levels were expressed relative to control astrocyte-conditioned medium.

### 2.16. Culture and Differentiation of LUHMES Cells into Human Postmitotic Neurons

Lund human mesencephalic cells (LUHMES; ATCC, Manassas, VA, USA, CRL-2927) were maintained under proliferative conditions in DMEM/F-12 medium supplemented with 2 mM L-glutamine, 1× N2 supplement, and 20 ng/mL recombinant human basic fibroblast growth factor (bFGF). Cells were cultured in flasks coated with 50 µg/mL poly-L-ornithine (Sigma-Aldrich), 1 µg/mL fibronectin (Sigma-Aldrich), and Geltrex basement membrane matrix (Thermo Fisher Scientific). Cultures were maintained at 37 °C in a humidified atmosphere containing 5% CO_2_. The culture medium was replaced every 2 days, and cells were passaged before reaching complete confluence.

When cultures reached approximately 70% confluence, neuronal differentiation was initiated by replacing the proliferation medium with differentiation medium consisting of DMEM/F-12 supplemented with 2 mM L-glutamine, 1× N2 supplement, 1 µg/mL tetracycline, and 2 ng/mL recombinant human glial cell line-derived neurotrophic factor (GDNF; R&D Systems, Minneapolis, MN, USA). Cells were maintained in differentiation medium for 2 days, then detached using trypsin and replated onto culture plates coated with poly-L-ornithine, fibronectin and Geltrex. The replated cells were maintained in differentiation medium for an additional 3 days, resulting in a total differentiation period of 5 days. At the end of differentiation, LUHMES cells displayed a postmitotic neuronal phenotype characterized by extensive neurite formation and expression of βIII-tubulin, microtubule-associated protein 2 (MAP2), PSD-95 and synaptophysin. Experimental treatments were performed after completion of the differentiation period. The culture and differentiation procedures were adapted from previously established LUHMES protocols [21,22].

### 2.17. MitoTracker Labeling of Human Astrocytes and Neurons

To label astrocyte-derived mitochondrial material, human astrocytes were incubated with 500 nM MitoTracker Red CMXRos (Thermo Fisher Scientific, cat. M7512) for 1 h at 37 °C. After incubation, the cultures were thoroughly washed with prewarmed culture medium to remove residual unbound probe, and fresh medium was added. Astrocytes were then maintained for an additional 24 h to generate conditioned medium containing fluorescently labeled mitochondrial material released by the cells. The conditioned medium was subsequently collected and applied to differentiated LUHMES-derived neurons.

For assessment of the neuronal mitochondrial membrane potential, differentiated LUHMES-derived neurons were incubated with 100 nM MitoTracker Deep Red FM (Thermo Fisher Scientific, cat. M22426) for 30 min at 37 °C. The cells were then washed to remove excess probe and fixed with 4% paraformaldehyde in phosphate-buffered saline for 15 min at room temperature. After fixation, the cultures were washed with phosphate-buffered saline and processed for fluorescence imaging and quantitative analysis.

### 2.18. In-Cell Western Analysis of Mitochondrial Dynamics Proteins

Protein levels were assessed by In-Cell Western analysis. Human astrocytes were cultured in black, clear-bottom 96-well plates and subjected to the indicated experimental treatments. At the end of the treatment period, cells were fixed with 4% paraformaldehyde in PBS for 20 min at room temperature. After fixation, cells were washed three times with PBS and permeabilized with 0.1% Triton X-100 in PBS for 10 min at room temperature. Non-specific antibody binding was blocked by incubating the cells with 1% bovine serum albumin in PBS for 1 h at room temperature. Cells were incubated overnight at 4 °C with primary antibodies against mitofusin 1 and mitofusin 2 (mouse anti-MFN1+2, 1:100; Abcam) and phosphorylated dynamin-related protein 1 at serine 616 (rabbit anti-p-DRP1 Ser616, 1:100; Thermo Fisher Scientific), diluted in blocking solution.

Following primary antibody incubation, cells were washed three times with PBS containing 0.1% Tween 20 and incubated with IRDye 800CW anti-rabbit or anti-mouse secondary antibodies, as appropriate, diluted 1:1000 in 1% BSA in PBS (LI-COR Biosciences, Lincoln, Nebraska, USA), for 1 h at room temperature in the dark. CellTag 700 Stain was included during the secondary antibody incubation to quantify total cellular protein in each well. Cells were then thoroughly washed with PBS containing 0.1% Tween 20, followed by a final wash with PBS. Plates were scanned using an Odyssey infrared imaging system (LI-COR Biosciences, Lincoln, Nebraska, USA). Fluorescence signals corresponding to MFN1/MFN2 and p-DRP1(Ser616) were quantified using U-SCAN-IT Gel software, version 6.1 (Silk Scientific Corp., Orem, UT, USA), for each well and normalized to the CellTag 700 total protein signal. Results were expressed relative to the untreated control group. Each experimental condition was analyzed in technical replicates, and the mean value of the replicates was used to represent each independent astrocyte culture.

### 2.19. Cell Viability Assessment (LDH)

We determined cellular cytotoxicity by measuring the extracellular activity of lactate dehydrogenase (LDH), which is an indicator of cellular injury. The LDH activity assay was performed using 50 μL of culture medium following the manufacturer’s instructions (CytoTox-Glo™ Cytotoxicity Assay, Promega Corporation, Madison, WI, USA, Cat. G1780).

### 2.20. Data and Statistical Analysis

Statistical analysis of quantitative data was performed using GraphPad Prism software version 8.0 (GraphPad Software, La Jolla, CA, USA). A 95% confidence interval was applied, and *p*-values less than 0.05 were considered statistically significant. Results are presented as mean ± SEM. For comparisons between two groups, Student’s *t*-test was used. For comparisons involving more than two groups or experimental conditions, one-way ANOVA followed by Bonferroni’s multiple-comparisons post hoc test was used. Detailed information regarding the statistical tests, *p*-values, and sample sizes is provided in the corresponding figure legends.

## 3. Results

### 3.1. Senescence Induces Mitochondrial Fragmentation and Ultrastructural Damage in Human Astrocytes

Cellular senescence is known to induce profound alterations in mitochondrial dynamics and function. However, these changes remain poorly characterized in human astrocytes. In murine astrocytes, mitochondrial fragmentation has been reported in models of senescence induced by cytosine arabinoside and maintained through long-term cultures [12,13]. More recently, we described an approach to induce senescence in both murine and human astrocytes using doxorubicin, a chemotherapeutic agent that induces DNA damage and activates canonical senescence pathways [9].

**Figure 1:**
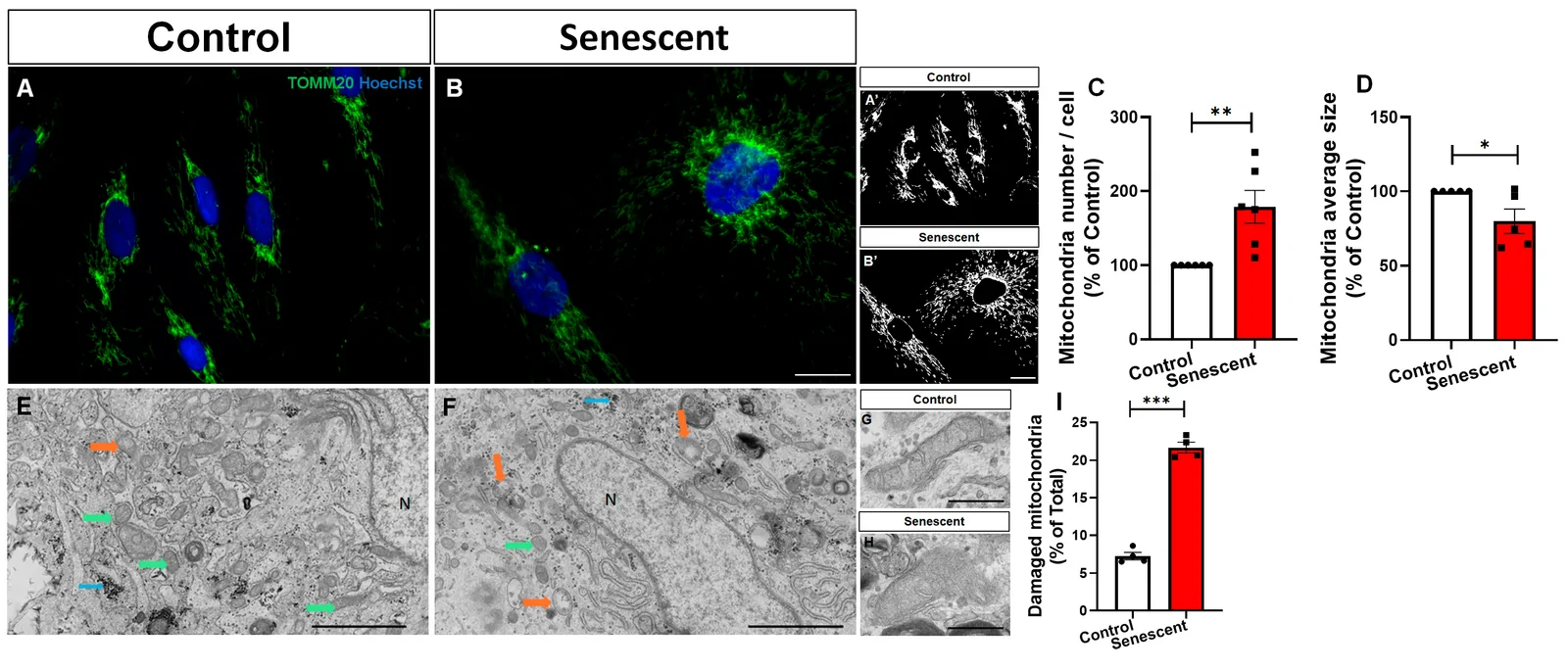
Senescent human astrocytes exhibit mitochondrial fragmentation and ultrastructural damage. (**A**,**B**) Representative fluorescence images of control and senescent astrocytes immunolabeled for TOMM20 (green); nuclei were counterstained with Hoechst 33342 (blue). (**A′**,**B′**) Representative binary masks used for mitochondrial morphometric analysis. (**C**) Number of TOMM20-positive mitochondrial particles per cell. (**D**) Mean mitochondrial particle size. (**E**,**F**) Representative transmission electron microscopy images of control and senescent astrocytes. Green arrows indicate mitochondria with preserved morphology, orange arrows indicate structurally altered mitochondria, and blue arrows indicate glycogen granules, a characteristic feature of the astrocytic cytoplasm. N indicates the nucleus. (**G**,**H**) Higher-magnification images showing representative mitochondrial ultrastructure. (**I**) Percentage of mitochondria classified as damaged based on membrane integrity, cristae organization, matrix electron density and organelle morphology. Morphometric data were normalized to control values. Data are expressed as mean ± SEM. Each symbol represents an independent culture, with *n* = 3–6 cultures per group. Statistical significance was determined using *t*-test. * *p* < 0.05, ** *p* < 0.01, *** *p* < 0.001. Scale bars: 20 µm (**A**,**B** and corresponding masks **A′**,**B′**), 2 µm (**E**,**F**) and 400 nm (**G**,**H**).

To determine whether cellular senescence alters mitochondrial organization in human astrocytes, we first assessed mitochondrial morphology by immunocytochemical detection of the outer mitochondrial membrane protein TOMM20. Compared with control astrocytes, doxorubicin-induced senescent astrocytes exhibited a marked increase in TOMM20-positive mitochondrial density (Figure 1A–C). This increase was accompanied by a significant reduction in the average size of individual mitochondrial structures (Figure 1D), indicating a shift towards a more fragmented mitochondrial network.

Because changes in TOMM20 staining do not necessarily reflect mitochondrial integrity, we next examined mitochondrial ultrastructure by transmission electron microscopy. Control astrocytes displayed mitochondria with preserved membrane organization, electron-dense matrices and well-defined cristae (Figure 1E,G). In contrast, senescent astrocytes showed a higher proportion of structurally abnormal mitochondria, including organelles with swollen profiles, disrupted or poorly defined cristae, irregular morphology and reduced matrix density (Figure 1F,H). Quantitative analysis confirmed a significant increase in the percentage of damaged mitochondria in senescent astrocytes compared with controls (Figure 1I).

Together, these findings demonstrate that doxorubicin-induced senescence remodels the mitochondrial network of human astrocytes, increasing mitochondrial density while reducing organelle size and promoting the accumulation of ultrastructurally damaged mitochondria.

### 3.2. Senescent Human Astrocytes Display Simultaneous Activation of Mitochondrial Fusion and Fission Pathways

**Figure 2:**
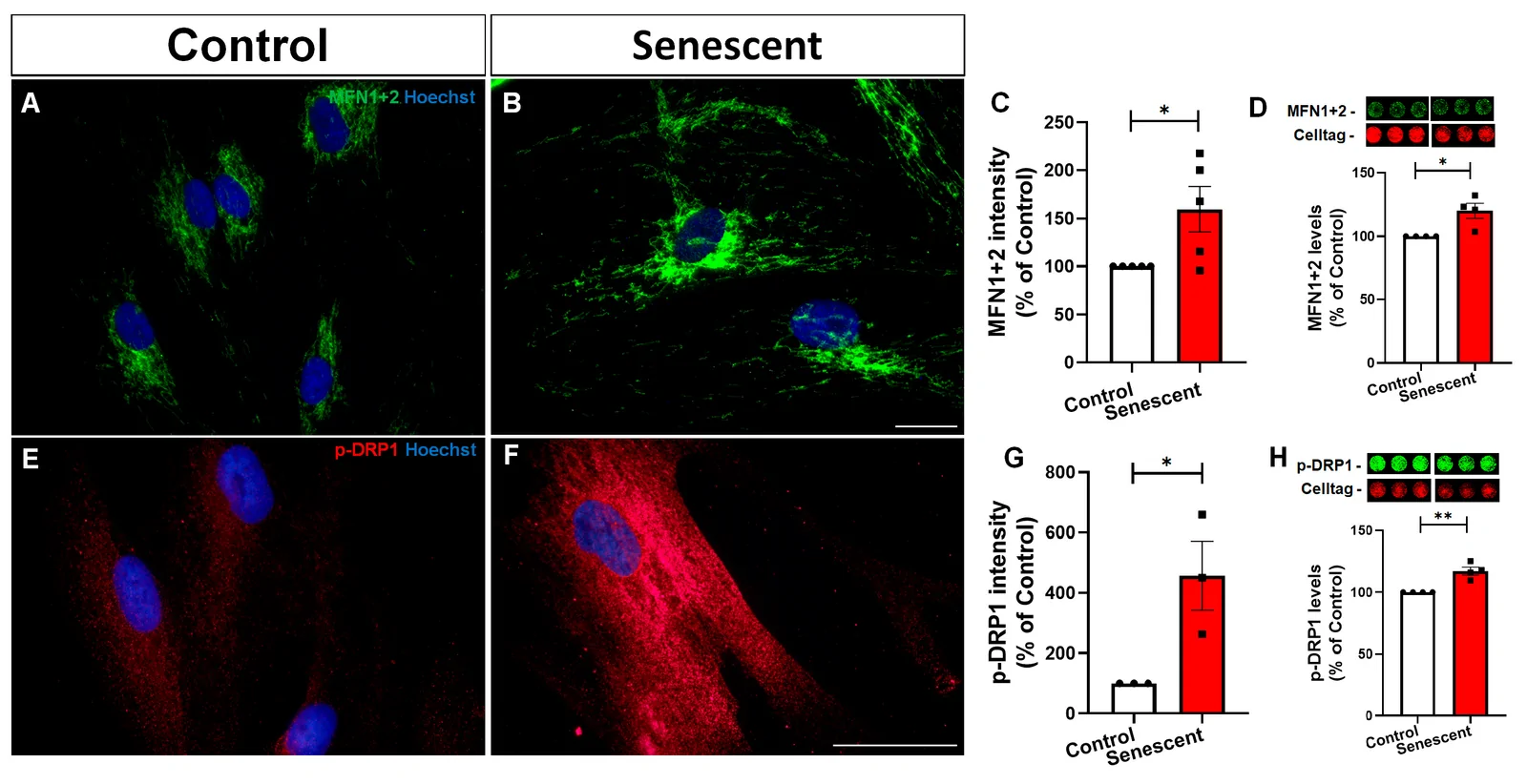
Senescent human astrocytes exhibit concurrent upregulation of mitochondrial fusion and fission proteins. (**A**,**B**) Representative fluorescence images of control and senescent astrocytes immunolabeled for mitofusin 1 and mitofusin 2 (MFN1+2, green); nuclei were counterstained with Hoechst 33342 (blue). (**C**) Quantification of MFN1+2 fluorescence intensity. (**D**) Representative In-Cell Western signals and quantification of MFN1+2 levels normalized to CellTag 700 total protein staining. (**E**,**F**) Representative fluorescence images of p-DRP1(Ser616) (red); nuclei were counterstained with Hoechst 33342 (blue). (**G**) Quantification of p-DRP1(Ser616) fluorescence intensity. (**H**) Representative In-Cell Western signals and quantification of p-DRP1(Ser616) levels normalized to CellTag 700 staining. Values were expressed relative to the control group. Data are mean ± SEM. Each symbol represents an independent culture, with *n* = 3–5 cultures per group. Statistical significance per group was determined using *t*-test. * *p* < 0.05, ** *p* < 0.01. Scale bars, 20 µm.

The coexistence of increased mitochondrial density and reduced mitochondrial size suggested an imbalance in the molecular machinery that regulates mitochondrial dynamics. We therefore evaluated key proteins involved in mitochondrial fusion and fission. Immunocytochemical analysis revealed increased staining intensity for mitofusin 1+2 (MFN1+2) in senescent astrocytes compared with control cells (Figure 2A–C). These findings were confirmed by in-cell western analysis, which showed higher levels of MFN1+2 in senescent astrocytes (Figure 2D). We next examined the activation of the mitochondrial fission protein dynamin-related protein 1 (DRP1) through phosphorylation at serine 616, a regulatory site associated with DRP1 activation, mitochondrial recruitment and the promotion of mitochondrial fission [23]. Senescent astrocytes exhibited increased p-DRP1(Ser616) immunoreactivity compared with control cells, with quantitative analysis confirming a significant elevation in staining intensity (Figure 2E–G). This result was validated by in-cell western analysis, which demonstrated increased p-DRP1(Ser616) levels in senescent astrocytes (Figure 2H). Thus, senescent human astrocytes exhibit concomitant upregulation of proteins associated with mitochondrial fusion and fission.

### 3.3. Mitochondrial Mass Markers Are Increased in Senescent Human Astrocytes

The increase in mitochondrial density observed in senescent astrocytes raised the possibility that mitochondrial biogenesis was enhanced as a compensatory response to organelle dysfunction, as previously reported by our group in a murine model of astrocyte aging [13]. To investigate this possibility, we assessed PGC-1α, a central transcriptional coactivator of mitochondrial biogenesis. PGC-1α coordinates this process in part by activating nuclear respiratory factor 1 (NRF1), which in turn promotes expression of mitochondrial transcription factor A (TFAM) and of nuclear-encoded mitochondrial proteins such as VDAC.

**Figure 3:**
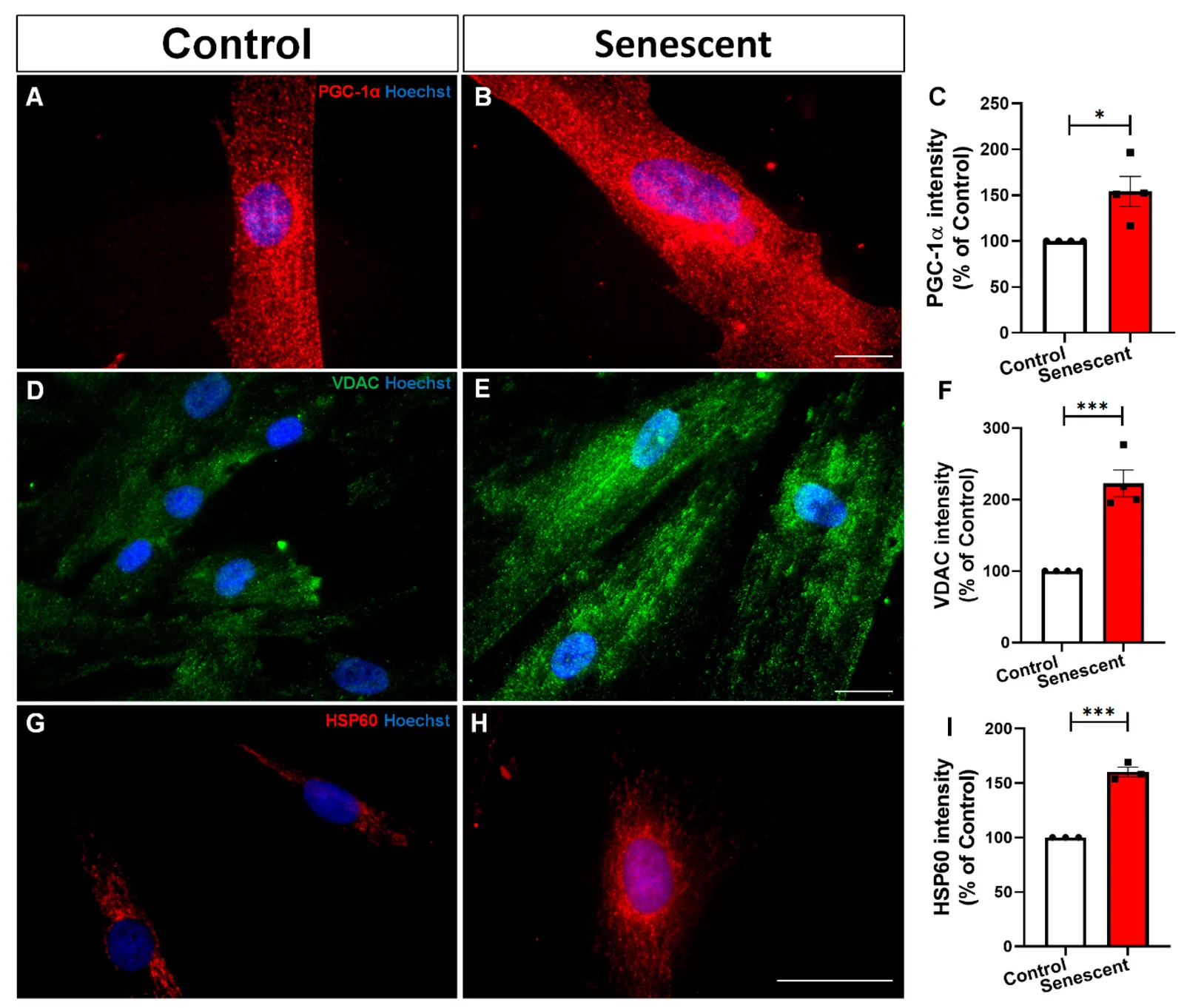
Senescent human astrocytes display increased PGC-1α, VDAC and HSP60 immunoreactivity. (**A**,**B**) Representative fluorescence images of control and senescent astrocytes immunolabeled for PGC-1α (red). (**C**) Quantification of PGC-1α fluorescence intensity. (**D**,**E**) Representative images of VDAC immunolabeling (green). (**F**) Quantification of VDAC fluorescence intensity. (**G**,**H**) Representative images of HSP60 immunolabeling (red). (**I**) Quantification of HSP60 fluorescence intensity. Nuclei were counterstained with Hoechst 33342 (blue). Fluorescence intensity was quantified per cell and expressed relative to the control group. Data are mean ± SEM from 3–4 independent cultures per group. Statistical significance was determined using *t*-test. * *p* < 0.05, *** *p* < 0.001. Scale bars, 20 µm.

Senescent astrocytes displayed a pronounced increase in PGC-1α immunoreactivity compared with control cells (Figure 3A–C). We next evaluated two additional markers associated with mitochondrial mass and organelle abundance. Immunocytochemical analysis showed increased expression of the outer mitochondrial membrane protein VDAC in senescent astrocytes (Figure 3D–F). Similarly, immunoreactivity for HSP60, a mitochondrial matrix chaperone involved in protein folding and mitochondrial proteostasis, was increased in senescent astrocytes (Figure 3G–I). Increased HSP60 levels are consistent with a compensatory response to mitochondrial proteotoxic stress and the accumulation of misfolded or damaged mitochondrial proteins. Together, these findings indicate that senescent human astrocytes accumulate mitochondrial proteins and activate stress-response pathways. 

### 3.4. Mitochondrial Accumulation in Senescent Astrocytes Is Accompanied by Bioenergetic Dysfunction

**Figure 4:**
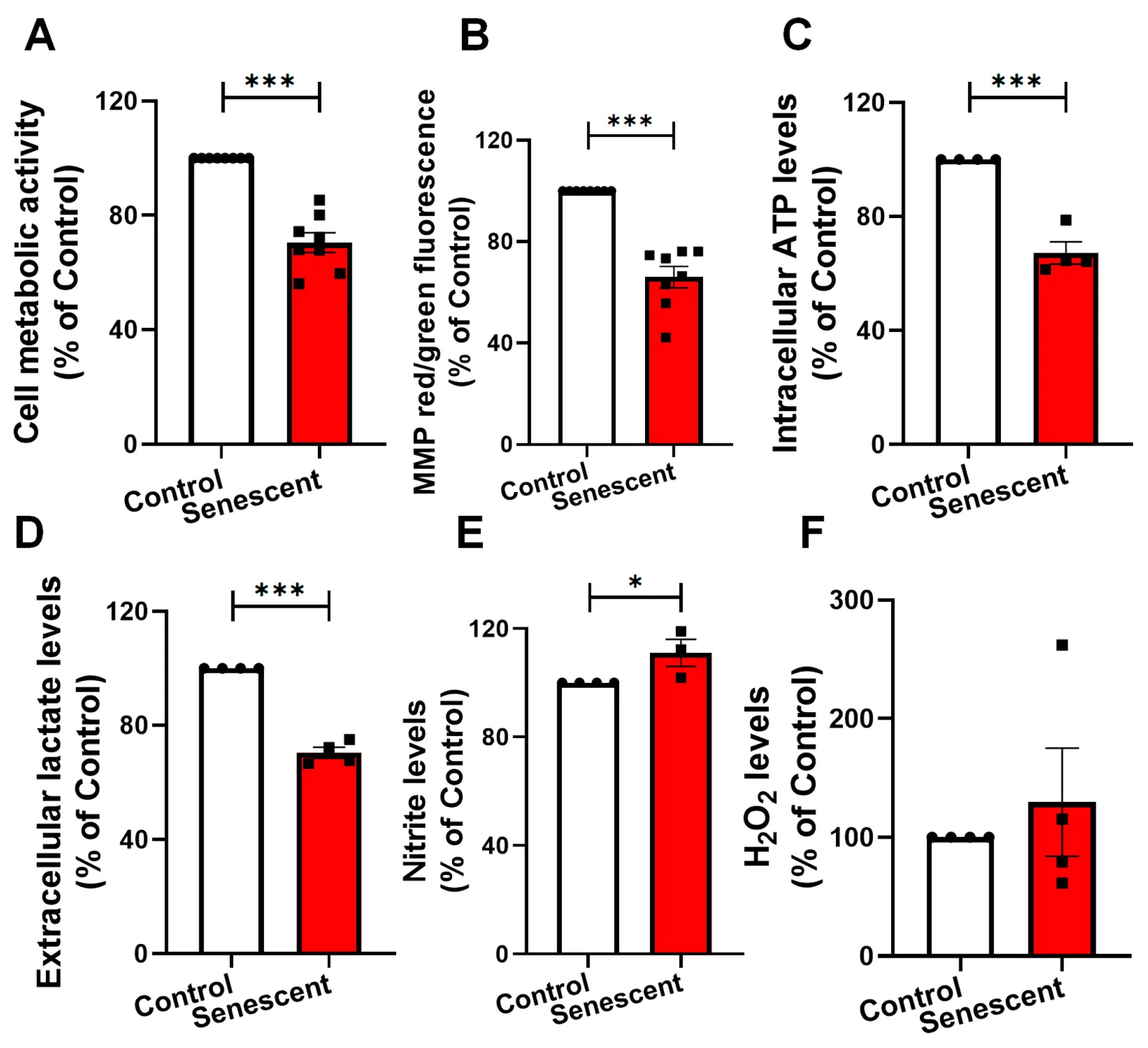
Senescent human astrocytes exhibit mitochondrial and metabolic dysfunction. (**A**) Cellular metabolic activity assessed by MTT reduction. (**B**) Mitochondrial membrane potential (MMP) assessed using the JC-1 red-to-green fluorescence ratio. (**C**) Intracellular ATP content measured using the CellTiter-Glo 2.0 luminescence assay. (**D**) Extracellular lactate levels. (**E**) Extracellular nitrite levels measured using the Griess reaction. (**F**) Extracellular hydrogen peroxide (H_2_O_2_) levels measured using the Amplex Red assay. Values were normalized to the corresponding control group. Data are mean ± SEM. Each symbol represents an independent culture, with *n* = 3–8 cultures per group depending on the assay. Statistical significance was determined using *t*-test. * *p* < 0.05, *** *p* < 0.001.

To determine whether the increased mitochondrial density and biogenesis-associated signaling observed in senescent astrocytes were associated with preserved mitochondrial function, we assessed multiple parameters of cellular metabolism, bioenergetics and redox homeostasis. Senescent astrocytes exhibited a significant reduction in MTT reduction capacity compared with control cells (Figure 4A), indicating decreased cellular metabolic activity. Consistent with this finding, JC-1 analysis revealed a marked loss of mitochondrial membrane potential, as demonstrated by a reduced aggregate-to-monomer fluorescence ratio (Figure 4B). Intracellular ATP levels were also significantly decreased in senescent astrocytes (Figure 4C), confirming impaired cellular energy availability.

Senescent astrocytes additionally displayed reduced extracellular lactate levels compared with control cells (Figure 4D), suggesting diminished glycolytic output or lactate release. Extracellular nitrite levels were significantly increased (Figure 4E), consistent with elevated nitric oxide production. Extracellular H_2_O_2_ levels were not significantly altered, although greater variability was observed in the senescent group (Figure 4F).

Collectively, these findings demonstrate that the increased mitochondrial density observed in senescent human astrocytes does not reflect improved mitochondrial competence. Instead, senescent astrocytes accumulate structurally abnormal and bioenergetically inefficient mitochondria, accompanied by reduced cellular metabolic activity, mitochondrial depolarization, ATP depletion and impaired lactate production or release.

### 3.5. Senescent Human Astrocytes Release Fewer and Bioenergetically Compromised Extracellular Mitochondrial Particles

Astrocytes can release mitochondria and mitochondrial material into the extracellular environment, establishing a form of intercellular metabolic communication with neurons. Previous studies have shown that the transfer of functional astrocyte-derived mitochondria can enhance neuronal bioenergetics and survival under conditions of ischemic, oxidative or chemotherapeutic stress [17,18,24]. Importantly, the biological outcome of this communication appears to depend on the functional competence of the released mitochondria [25].

We therefore investigated whether primary human astrocytes release extracellular mitochondrial particles and further characterized the particulate fraction present in conditioned medium. Conditioned medium from control astrocytes was passed through a 0.22 µm filter to generate mitochondria-depleted conditioned medium (mdCM), and both fractions were stained with MitoTracker Green and analyzed by flow cytometry. Filtration markedly reduced the number of MitoTracker Green-positive events, indicating that most of the detected mitochondrial-associated signal was contained within a filter-retained particulate fraction (Supplementary Figure S1A–D). To further assess whether extracellular ATP was associated with sedimentable material, conditioned medium was sequentially centrifuged and the 20,000× *g* pellet and corresponding supernatant were analyzed separately (Supplementary Figure S1E). ATP was strongly enriched in the 20,000× *g* pellet compared with both the starting conditioned medium and the corresponding supernatant (Supplementary Figure S1F), supporting the presence of ATP-containing extracellular particulate material enriched in mitochondrial components.

**Figure 5:**
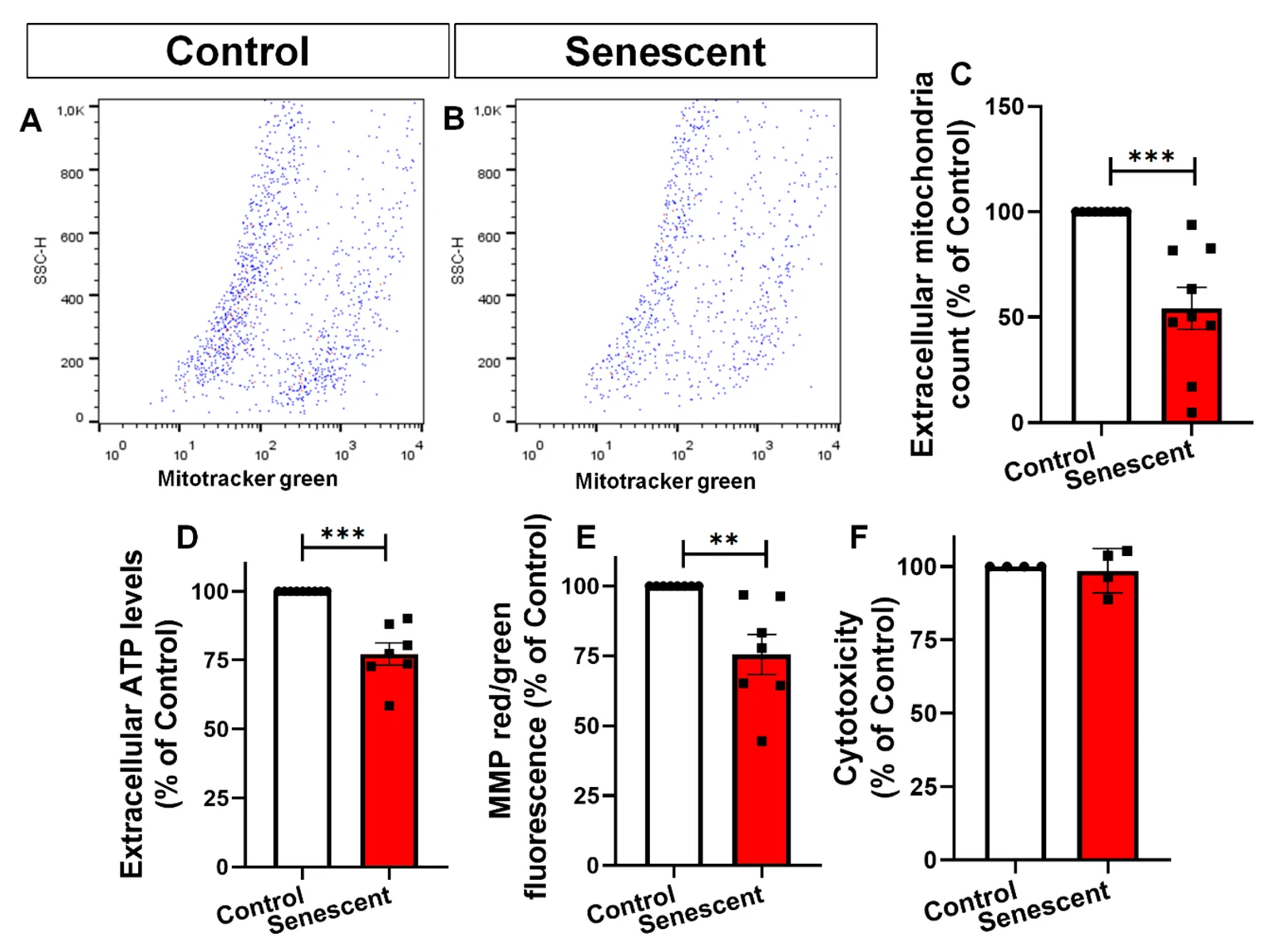
Astrocyte senescence reduces the abundance and polarization of extracellular mitochondrial particles. Conditioned medium was collected over 24 h from control and doxorubicin-induced senescent human astrocytes. (**A**,**B**) Representative flow-cytometry plots showing side scatter (SSC-H) and MitoTracker Green fluorescence in conditioned medium from control and senescent astrocytes. MitoTracker Green-positive events were classified as extracellular mitochondrial particles. (**C**) Quantification of extracellular mitochondrial particle number. (**D**) Extracellular ATP levels in astrocyte-conditioned medium. (**E**) Membrane potential of extracellular mitochondrial particles assessed using the JC-1 red-to-green fluorescence ratio. (**F**) Cytotoxicity in the donor astrocyte cultures assessed by extracellular lactate dehydrogenase activity. Measurements were expressed relative to control astrocyte-conditioned medium. Data are mean ± SEM. Each symbol represents an independent culture, with *n* = 4–9 cultures per group depending on the assay. Statistical significance was determined using *t*-test. ** *p* < 0.01, *** *p* < 0.001.

We next examined whether astrocyte senescence affected the abundance and functional state of the extracellular mitochondrial compartment. Conditioned medium from senescent astrocytes contained significantly fewer MitoTracker Green-positive particles than conditioned medium from control astrocytes (Figure 5A–C), indicating reduced extracellular release of mitochondrial material. Extracellular ATP levels were significantly decreased in conditioned medium from senescent astrocytes (Figure 5D). Assessment of mitochondrial membrane potential using JC-1 further revealed a lower aggregate-to-monomer fluorescence ratio in conditioned medium from senescent astrocytes (Figure 5E), consistent with reduced polarization of the extracellular mitochondrial particles. Importantly, extracellular LDH activity did not differ between control and senescent astrocyte cultures (Figure 5F), indicating that these changes were not attributable to overt plasma membrane damage or nonspecific release of intracellular contents.

Together, these findings demonstrate that senescence disrupts the extracellular mitochondrial compartment of primary human astrocytes by reducing both the abundance and the polarization of released mitochondrial particles. Rather than providing the functionally competent mitochondrial material previously associated with neuronal protection, senescent astrocytes release fewer and less polarized mitochondrial particles in a medium with lower ATP content.

### 3.6. Human Neurons Acquire Mitochondrial Material Derived from Human Astrocytes

To investigate how astrocyte senescence affects neuronal mitochondrial homeostasis, we used human postmitotic neurons derived from differentiated LUHMES cells. Before examining the effects of astrocyte-conditioned medium, we characterized the neuronal phenotype generated by the differentiation protocol. After 5 days of differentiation, LUHMES cells exhibited a postmitotic neuronal morphology characterized by an extensive neuritic network and expression of the neuronal markers βIII-tubulin and MAP2, together with the synaptic proteins synaptophysin and PSD-95 (Supplementary Figure S2). These findings confirmed the acquisition of a differentiated human neuronal phenotype suitable for investigating astrocyte-to-neuron metabolic communication.

To assess neuronal acquisition of astrocyte-derived mitochondrial labeling, control and senescent human astrocytes were incubated with MitoTracker Red CMXRos for 1 h, washed, and maintained in fresh medium for 24 h to generate conditioned medium containing fluorescently labeled mitochondrial material. The conditioned medium was then collected and applied to differentiated LUHMES-derived neurons for an additional 24 h.

MitoTracker-positive signal was detected within LUHMES-derived neurons exposed to astrocyte-conditioned medium, indicating neuronal acquisition of astrocyte-derived fluorescent material (Supplementary Figure S3B,C). Unexpectedly, neurons treated with conditioned medium from senescent astrocytes exhibited a higher MitoTracker-associated signal than those exposed to conditioned medium from control astrocytes (Supplementary Figure S3D). These observations indicate that material carrying the astrocytic mitochondrial label reaches recipient neurons under both conditions.

### 3.7. Senescent Astrocyte-Conditioned Medium Fails to Support Mitochondrial Homeostasis in Human Postmitotic Neurons

Astrocytes provide essential metabolic and trophic support to neurons through the release of soluble factors, metabolites and mitochondrial components [26]. We therefore investigated whether factors released by control human astrocytes modulated mitochondrial homeostasis in human postmitotic neurons, and whether this supportive effect was compromised by astrocyte senescence. Differentiated LUHMES-derived neurons were maintained for 24 h in non-conditioned medium (DMEM) or exposed to conditioned medium obtained from control astrocytes (CM Control) or senescent astrocytes (CM Senescent).

**Figure 6:**
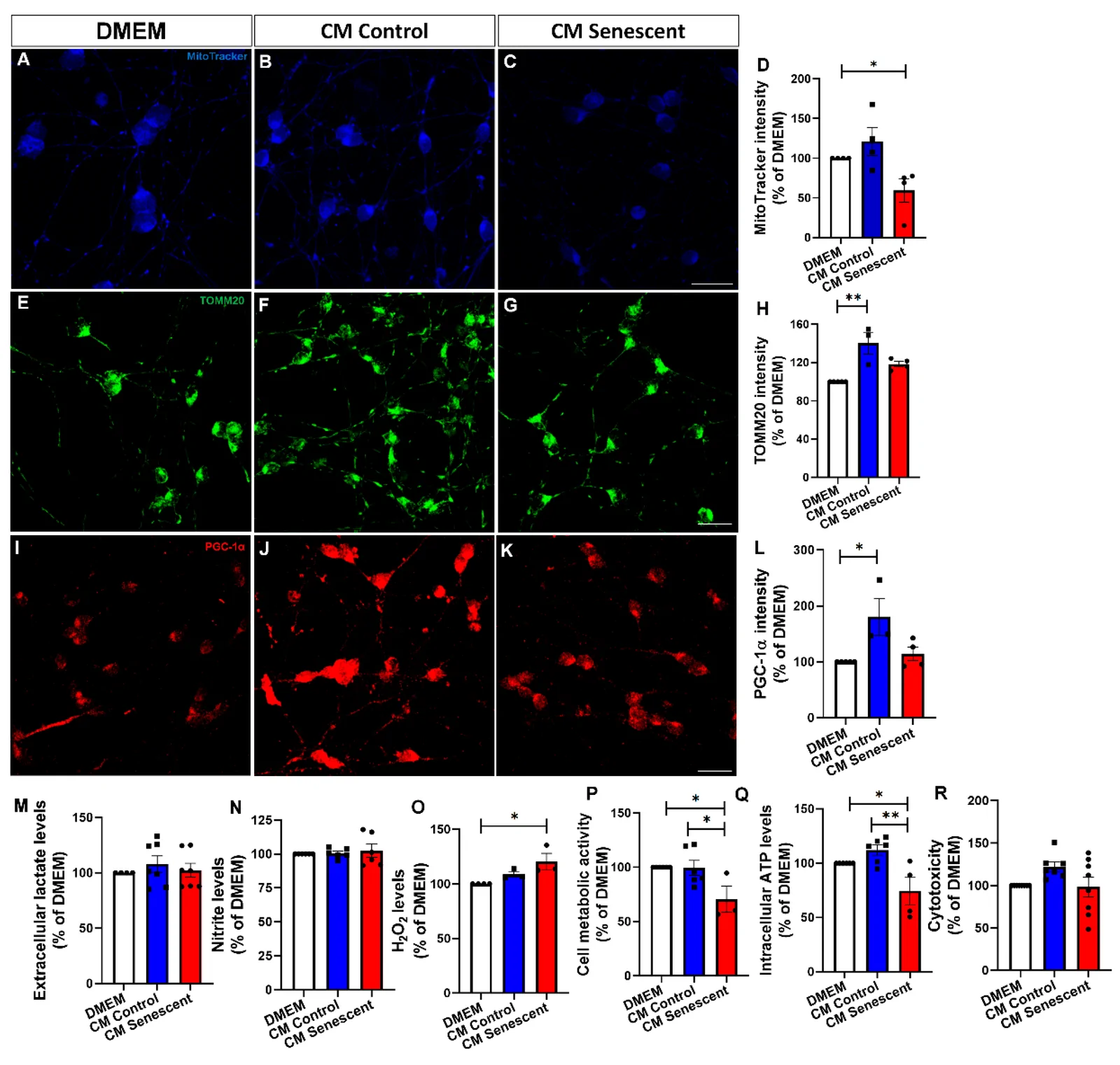
Conditioned medium from senescent astrocytes fails to sustain neuronal mitochondrial homeostasis and compromises neuronal bioenergetic function. Differentiated LUHMES-derived neurons were maintained for 24 h in non-conditioned DMEM/F12 medium or exposed to conditioned medium collected from control astrocytes (CM Control) or doxorubicin-induced senescent astrocytes (CM Senescent). (**A**–**C**) Representative images of neuronal MitoTracker Deep Red FM fluorescence; the far-red signal is displayed in blue. (**D**) Quantification of MitoTracker fluorescence intensity. (**E**–**G**) Representative images of TOMM20 immunolabeling. (**H**) Quantification of TOMM20 fluorescence intensity. (**I**–**K**) Representative images of PGC-1α immunolabeling. (**L**) Quantification of PGC-1α fluorescence intensity. (**M**) Extracellular lactate levels. (**N**) Extracellular nitrite levels. (**O**) Extracellular H_2_O_2_ levels. (**P**) Cellular metabolic activity assessed by MTT reduction. (**Q**) Intracellular ATP content. (**R**) Cytotoxicity assessed by extracellular lactate dehydrogenase activity. All fluorescence and biochemical measurements were expressed relative to neurons maintained in non-conditioned medium (% of DMEM). Data are presented as mean ± SEM. Each symbol represents an independent neuronal culture, with *n* = 3–8 cultures per group depending on the assay. Statistical significance was determined using one-way ANOVA followed by Bonferroni’s multiple-comparisons test. All possible pairwise comparisons were performed between the three experimental groups: DMEM vs. CM Control, DMEM vs. CM Senescent, and CM Control vs. CM Senescent. * *p* < 0.05, ** *p* < 0.01. Scale bars, 20 µm.

Because MitoTracker Deep Red accumulates in mitochondria in a membrane potential-dependent manner, its fluorescence intensity was used as an indirect indicator of neuronal mitochondrial membrane potential [27]. Human postmitotic neurons exposed to CM Senescent exhibited significantly reduced MitoTracker Deep Red fluorescence compared with neurons maintained in non-conditioned medium, consistent with mitochondrial depolarization (Figure 6A–D). Consistent with this observation, CM Control increased neuronal TOMM20 immunoreactivity relative to non-conditioned medium (Figure 6E–H), supporting an increase in mitochondrial content. CM Control also enhanced neuronal PGC-1α immunoreactivity (Figure 6I–L), indicating activation of mitochondrial biogenesis-associated signaling or metabolic adaptation in recipient neurons. In contrast, CM Senescent failed to induce the increases in TOMM20 and PGC-1α signals observed following exposure to CM Control.

Together, these findings demonstrate that factors released by non-senescent human astrocytes promote mitochondrial content and biogenesis-associated signaling in human postmitotic neurons. Astrocyte senescence abolishes this supportive response, revealing a reduced capacity of senescent astrocytes to sustain neuronal mitochondrial homeostasis.

### 3.8. Loss of Astrocytic Mitochondrial Support Is Associated with Redox and Bioenergetic Dysfunction in Human Postmitotic Neurons

Having established that conditioned medium from senescent astrocytes failed to promote mitochondrial homeostasis in human postmitotic neurons, we next examined whether this loss of support was accompanied by functional metabolic and redox alterations.

Extracellular lactate and nitrite levels did not differ significantly among neurons maintained in non-conditioned medium or exposed to CM Control or CM Senescent (Figure 6M,N). In contrast, human postmitotic neurons exposed to CM Senescent exhibited significantly increased extracellular H_2_O_2_ levels compared with neurons maintained in non-conditioned medium (Figure 6O), indicating enhanced oxidative stress.

This redox imbalance was accompanied by a marked reduction in MTT reduction capacity in neurons treated with CM Senescent compared with both the non-conditioned medium and CM Control groups (Figure 6P), indicating impaired neuronal metabolic activity. Intracellular ATP levels were also reduced in human postmitotic neurons exposed to CM Senescent, particularly compared with neurons treated with CM Control (Figure 6Q), further demonstrating compromised bioenergetic homeostasis. Importantly, extracellular LDH activity remained unchanged across the experimental groups (Figure 6R), indicating that the reductions in metabolic activity and ATP availability occurred in the absence of detectable plasma membrane damage or overt cytotoxicity.

Collectively, these findings support the concept that factors released by senescent astrocytes into the conditioned medium may contribute to early oxidative and bioenergetic dysfunction in human postmitotic neurons before the onset of overt cytotoxicity.

## 4. Discussion

In this study, we identify mitochondrial dysfunction as a central component of senescence in primary human astrocytes and show that this phenotype is accompanied by a loss of metabolic support to human postmitotic neurons. Doxorubicin-induced senescent astrocytes accumulated a denser population of smaller and ultrastructurally damaged mitochondria, together with increased expression of proteins associated with mitochondrial fission, fusion, biogenesis-related signalling, and proteostasis. Despite this apparent expansion and remodelling of the mitochondrial compartment, senescent astrocytes exhibited reduced mitochondrial membrane potential, ATP content, and cellular metabolic activity. Senescence also altered the extracellular mitochondrial compartment, reducing the abundance and bioenergetic competence of released mitochondrial particles. At the functional level, conditioned medium from control astrocytes increased neuronal TOMM20 and PGC-1α, whereas conditioned medium from senescent astrocytes failed to elicit these responses and instead induced H_2_O_2_ accumulation, ATP depletion, and reduced MTT reduction without detectable LDH release. These findings support a model in which astrocyte senescence precedes overt neuronal death by creating a paracrine environment that withdraws mitochondrial support and promotes early neuronal redox and bioenergetic stress.

The intracellular mitochondrial phenotype extends previous observations from murine models of astrocyte ageing and senescence. Long-term differentiated murine astrocytes display mitochondrial fragmentation, accumulation of damaged organelles, and defective mitophagy [12], whereas aged astrocytes exhibit simultaneous activation of fission, fusion, and biogenesis-related pathways despite persistent mitochondrial dysfunction [13]. The present study extends these findings to primary human astrocytes using a previously characterized doxorubicin model that produces stable cell-cycle arrest, DNA damage signaling, and a pro-inflammatory senescence-associated secretory phenotype, SASP [9]. Thus, mitochondrial remodelling appears not to be restricted to prolonged murine culture but may represent a conserved feature of the senescent astrocyte state.

Similar mitochondrial alterations have been reported in other human astrocyte senescence models. Clusterin deficiency induced mitochondrial depolarisation, oxidative stress, increased mitochondrial mass, and reduced ATP production in human astrocytic cells [28]. Likewise, long-term cultured human pluripotent stem cell-derived astrocytes exhibited reduced mitochondrial membrane potential, altered mitochondrial morphology, and diminished support for neuronal differentiation and survival [29]. These findings support mitochondrial dysfunction and loss of neuronal support as recurrent features of human astrocyte senescence.

The concurrent increase in MFN1/2 and DRP1 phosphorylation at Ser616 indicates broad engagement of the mitochondrial dynamics machinery. DRP1 phosphorylation at Ser616 favours its recruitment to mitochondria and promotes fission [23], but increased mitofusin abundance does not necessarily demonstrate effective mitochondrial fusion. In the presence of reduced mitochondrial size and extensive cristae disruption, the combined elevation of fission- and fusion-associated proteins is more consistent with an unsuccessful attempt to remodel a damaged mitochondrial network.

A similar interpretation applies to the increases in PGC-1α, VDAC, TOMM20, and HSP60. PGC-1α coordinates mitochondrial biogenesis-related transcription, whereas VDAC and TOMM20 are components of the outer mitochondrial membrane. HSP60 is a mitochondrial matrix chaperone that contributes to protein folding and maintenance of mitochondrial proteostasis [30]. Its increase is therefore compatible with activation of a stress response to mitochondrial protein damage. Consistent with the importance of this pathway, HSP60 deficiency induces mitochondrial dysfunction and cellular senescence in astrocytes [31]. Increased HSP60 in our model may reflect a compensatory proteostatic response that is insufficient to restore mitochondrial function.

The accumulation of PGC-1α, VDAC, TOMM20, and HSP60 suggests expansion of mitochondrial components and activation of stress-adaptive pathways. However, these markers do not independently establish productive mitochondrial biogenesis. The persistence of mitochondrial depolarisation, ATP depletion, and reduced cellular metabolic activity indicates that this compensatory response is insufficient to restore bioenergetic competence, potentially because the production of mitochondrial components is not adequately coupled to mitophagy and organelle turnover.

Astrocyte senescence also produced a broader metabolic and redox phenotype. Reduced extracellular lactate suggests impaired glycolytic output or lactate release, potentially limiting an important component of astrocyte-to-neuron metabolic cooperation. Astrocytic glutamate uptake stimulates glycolysis and lactate production, coupling neuronal activity to local energy metabolism [32], and neuron–astrocyte metabolic interactions are central to the maintenance of cerebral energy homeostasis [33]. Thus, reduced lactate availability may contribute to the impaired neuronal support associated with astrocyte senescence.

The importance of astrocytic metabolic competence is further supported by studies showing that healthy astrocytes restore mitochondrial function and dynamics in human iPSC-derived dopaminergic neurons exposed to respiratory-chain inhibitors [16]. Accordingly, the increases in neuronal TOMM20 and PGC-1α induced by control astrocyte-conditioned medium may represent an active component of physiological astrocyte-to-neuron mitochondrial support. Increased extracellular nitrite further indicates enhanced nitrosative stress, whereas extracellular H_2_O_2_ was not significantly altered in donor astrocyte cultures. These changes are relevant because astrocytic mitochondrial dysfunction can precede loss of viability and compromise glutamate handling and neuronal protection [34]. In human cellular models, mitochondrial complex I dysfunction also enhances astrocyte inflammatory responsiveness and negatively affects neuronal physiology during cytokine exposure [15].

A major limitation of this study is that the association between alterations in the extracellular mitochondrial compartment and neuronal dysfunction does not establish causality. Soluble SASP factors and other components of the senescent astrocyte secretome may also contribute to the observed neuronal phenotype, indicating that astrocyte senescence may compromise neuronal support through mechanisms extending beyond mitochondrial metabolism. Consistent with this possibility, senescent human astrocytes develop a pro-inflammatory SASP, exhibit reduced expression of glutamate and potassium transporters, and show a diminished capacity to protect neurons against excitotoxicity [7]. In a complementary human model, rotenone-induced astrocyte senescence generated a senescent secretome that exacerbated degeneration of iPSC-derived midbrain neurons carrying an SNCA locus duplication [35]. Together, these findings support the concept that astrocyte senescence and mitochondrial stress generate a complex paracrine environment capable of increasing neuronal vulnerability. Future studies directly comparing the effects of complete and mitochondria-depleted conditioned media will therefore be required to determine the specific contribution of extracellular mitochondrial particles to the neuronal phenotype observed here.

Importantly, doxorubicin was removed after senescence induction, followed by extensive washing and at least three complete medium changes before conditioned-medium collection. This procedure substantially minimizes the possibility of doxorubicin carryover, making it unlikely that the neuronal alterations observed after conditioned-medium exposure were driven by residual drug rather than by factors released by senescent astrocytes. Other models have shown that senescent astrocytes reduce neuronal mitochondrial membrane potential, redox balance, and mitochondrial mass [36]. The neuronal phenotype observed here is therefore unlikely to result from a single signalling pathway or toxic factor. Instead, it may arise from convergent alterations in metabolites, neurotrophic support, inflammatory mediators, extracellular vesicles, and extracellular mitochondrial communication.

Intercellular mitochondrial exchange has emerged as a potential component of neuroglial communication. Following cerebral ischaemia, astrocytes release functional mitochondria that enter damaged neurons and promote survival through a CD38-dependent pathway [17]. Astrocyte-to-neuron mitochondrial transfer also improves neuronal health after cisplatin-induced mitochondrial injury [24], whereas astrocytic LRP1 promotes mitochondrial transfer and limits ischaemic brain injury [18]. Moreover, transplantation of mitochondria from healthy astrocytes into injured astrocytes restored mitochondrial function and enhanced AMPK/PGC-1α signalling [37]. Although this response was demonstrated in astrocytes rather than neurons, it supports the possibility that extracellular mitochondrial communication may contribute to the neuronal PGC-1α response induced by control astrocyte-conditioned medium.

The biological consequences of extracellular mitochondrial communication are likely to depend on the quality of the released material. Reduced mitochondrial O-GlcNAcylation produces extracellular astrocytic mitochondria with lower membrane potential and mtDNA content and diminishes their neuroprotective capacity [25]. Conversely, fragmented and dysfunctional mitochondria released by microglia can induce neurotoxic astrocyte responses and propagate inflammatory neurodegeneration [38]. Extracellular mitochondria have also been reported to activate microglia and promote neuroinflammation after traumatic brain injury [39]. These findings indicate that mitochondrial release should not be regarded as intrinsically protective or detrimental; its biological consequences are determined by donor-cell state, mitochondrial quality, recipient-cell demand, and the route of transfer.

Our findings add astrocyte senescence to this framework. Primary human astrocytes released MitoTracker Green-positive extracellular particles with JC-1 signals consistent with membrane polarisation and detectable ATP-containing material. Senescent astrocytes released fewer particles, with a lower JC-1 aggregate-to-monomer ratio and reduced extracellular ATP. These findings indicate that senescence disrupts both the abundance and bioenergetic competence of the extracellular mitochondrial compartment. They do not, however, demonstrate that these particles directly cause the neuronal dysfunction observed after exposure to senescent astrocyte-conditioned medium. The reduced release of bioenergetically competent mitochondrial material may primarily represent the loss of a physiological astrocyte-mediated support mechanism. A non-mutually exclusive possibility is that damaged mitochondrial components contribute to extracellular stress signalling.

Astrocyte-derived MitoTracker-associated fluorescence was detected in human postmitotic neurons exposed to conditioned medium from both control and senescent astrocytes. The stronger neuronal signal following exposure to senescent astrocyte-conditioned medium should be interpreted cautiously. It may reflect increased uptake of astrocyte-derived mitochondrial fragments or vesicles, impaired clearance of dysfunctional mitochondrial material, or both. However, these possibilities remain hypothetical because mitochondrial uptake kinetics and autophagic or lysosomal flux were not directly evaluated. Thus, increased MitoTracker-associated fluorescence may indicate intracellular retention of mitochondrial material rather than successful mitochondrial incorporation or functional rescue.

The strongest evidence for neuronal dysfunction derives from the response of human postmitotic neurons to the complete astrocyte secretome. Conditioned medium from control astrocytes increased neuronal TOMM20 and PGC-1α, consistent with increased mitochondrial protein abundance and activation of biogenesis-related signalling. This adaptive response was lost with senescent astrocyte-conditioned medium, which instead increased neuronal H_2_O_2_ and reduced ATP availability and cellular metabolic activity without increasing extracellular LDH. Together, these changes define an early, sublethal state of neuronal mitochondrial stress, characterized by loss of the TOMM20/PGC-1α response, H_2_O_2_ accumulation, ATP depletion, and decreased metabolic activity in the absence of detectable cytotoxicity (LDH release).

Although LUHMES cells are mesencephalic-derived, our differentiation protocol does not include db-cAMP and generates a broad postmitotic neuronal phenotype rather than a fully dopaminergic population. These cells express neuronal and synaptic markers and display electrophysiological activity, supporting their use for evaluating general neuronal responses to astrocyte-conditioned medium [21]. Nevertheless, the combination of LUHMES-derived neurons with cortical astrocytes does not fully reproduce region-specific neuroglial interactions, and this limitation should be considered when generalizing the present findings to distinct neuronal populations and brain regions.

This neuronal redox phenotype is consistent with previous observations that senescent rodent astrocytes reduce neuronal mitochondrial membrane potential and mitochondrial mass and decrease the neuronal GSH/GSSG ratio [36]. Impaired glutathione-dependent redox buffering may therefore contribute to the H_2_O_2_ accumulation observed in human postmitotic neurons in our model. Because neuronal excitability, synaptic transmission, calcium handling, proteostasis, and mitochondrial quality control are energetically demanding, sustained ATP deficiency and oxidative stress may progressively reduce neuronal resilience and amplify vulnerability to subsequent pathological insults. Together, these findings indicate that astrocyte senescence compromises neuronal mitochondrial homeostasis before overt neuronal death becomes detectable, supporting the concept that dysfunctional astrocytes actively shape early stages of brain ageing and neurodegeneration. In addition, astrocyte-conditioned medium contains SASP factors, metabolites, extracellular vesicles, and mitochondrial particles, precluding attribution of the neuronal phenotype to a single component. The relative contribution of each of these factors to neuronal dysfunction remains to be determined and should be addressed in future mechanistic studies.

## 5. Conclusions

Overall, our findings identify astrocyte senescence as an upstream disruptor of mitochondrial homeostasis within the human neuroglial unit. Senescent astrocytes accumulate structurally damaged and bioenergetically inefficient mitochondria, release fewer bioenergetically competent extracellular mitochondrial particles, and generate a paracrine environment that fails to support mitochondrial adaptation in human postmitotic neurons. Rather than attributing neuronal dysfunction to a single mediator, our results support a multifactorial model involving loss of metabolic and trophic support, SASP-associated signalling, and altered extracellular mitochondrial communication. This early withdrawal of astrocytic support may establish a permissive state for progressive neuronal dysfunction during brain ageing and neurodegenerative disease.

## Data Availability

Data will be made available on request.

## Supplementary Materials

The following supporting information can be downloaded at: https://www.mdpi.com/article/10.3390/antiox15091127/s1, Figure S1. Filtration and differential centrifugation characterize extracellular mitochondrial-associated material in human astrocyte-conditioned medium. (A) Schematic representation of the generation of mitochondria-depleted conditioned medium. Conditioned medium (CM) collected from primary human astrocytes was passed through a sterile 0.22-µm syringe filter to generate mitochondria-depleted conditioned medium (mdCM). Samples were incubated with MitoTracker Green and analyzed by flow cytometry. (B, C) Representative flow-cytometry plots showing side scatter (SSC-H) and MitoTracker Green fluorescence in unfiltered CM and mdCM. (D) Quantification of MitoTracker Green-positive extracellular particles, expressed relative to unfiltered CM. (E) Schematic representation of the differential centrifugation protocol used to fractionate astrocyte-conditioned medium. Following removal of cellular debris at 2,000 × g for 10 min, the resulting supernatant was centrifuged at 20,000 × g for 30 min to obtain a particulate pellet and the corresponding supernatant. (F) ATP content measured in the indicated conditioned-medium fractions. Data are presented as mean ± SEM. In (D), statistical significance was determined using a *t*-test comparing CM and mdCM. In (F), statistical significance was assessed by one-way ANOVA followed by Bonferroni’s multiple-comparisons test, with pairwise comparisons performed between homogenate and supernatant, homogenate and pellet, and supernatant and pellet. *** *p* < 0.001. Figure S2. Differentiation and phenotypic characterization of human LUHMES-derived neurons. (A) Schematic representation of the LUHMES differentiation protocol. Proliferating LUHMES progenitor cells were maintained in differentiation medium containing tetracycline (1 µg/mL) and glial cell line-derived neurotrophic factor (GDNF, 2 ng/mL) for 2 days. Cells were then trypsinized, replated and maintained in differentiation medium for an additional 3 days, resulting in a total differentiation period of 5 days. (B) Representative phase-contrast image showing the morphology and extensive neuritic network of differentiated LUHMES cells. (C–F) Representative fluorescence images showing expression of microtubule-associated protein 2 (MAP2, C), βIII-tubulin (D), synaptophysin (E) and postsynaptic density protein 95 (PSD-95, F). Scale bars, 20 µm. Figure S3. Human LUHMES-derived neurons acquire MitoTracker-labeled mitochondrial material released by human astrocytes. (A) Schematic representation of the experimental protocol. Control and doxorubicin-induced senescent astrocytes were labeled with MitoTracker Red CMXRos for 1 h, thoroughly washed, and maintained in fresh medium for 24 h. Conditioned medium from control astrocytes (CM Control) or senescent astrocytes (CM Senescent) was subsequently collected and applied to differentiated LUHMES-derived neurons for 24 h. (B, C) Representative fluorescence images showing astrocyte-derived MitoTracker signal in neurons exposed to CM Control or CM Senescent. (D) Quantification of MitoTracker fluorescence intensity in recipient neurons, expressed relative to the CM Control group. Data are mean ± SEM from five independent cultures per group. Statistical significance was determined using *t*-test. * *p* < 0.05. Scale bars, 20 μm.

## Abbreviations

The following abbreviations are used in this manuscript:

CM	Conditioned medium
DMEM	Dulbecco’s Modified Eagle Medium
DRP1	Dynamin-related protein 1
H_2_O_2_	Hydrogen peroxide
HSP60	Heat shock protein 60
LDH	Lactate dehydrogenase
LUHMES	Lund human mesencephalic cells
mdCM	Mitochondria-depleted conditioned medium
MFN1	Mitofusin 1
MFN2	Mitofusin 2
MMP	Mitochondrial membrane potential
MTT	3-(4,5-Dimethylthiazol-2-yl)-2,5-diphenyltetrazolium bromide
NO	Nitric oxide
PGC-1α	Peroxisome proliferator-activated receptor gamma coactivator 1-alpha
p-DRP1	Phosphorylated dynamin-related protein 1
ROS	Reactive oxygen species
SASP	Senescence-associated secretory phenotype
TOMM20	Translocase of outer mitochondrial membrane 20
VDAC	Voltage-dependent anion channel

## References

[1] Attwell D., Laughlin S.B. (2001). An energy budget for signaling in the grey matter of the brain. J. Cereb. Blood Flow Metab. Off. J. Int. Soc. Cereb. Blood Flow Metab..

[2] Sofroniew M.V., Vinters H.V. (2010). Astrocytes: Biology and pathology. Acta Neuropathol..

[3] Habib N., McCabe C., Medina S., Varshavsky M., Kitsberg D., Dvir-Szternfeld R., Green G., Dionne D., Nguyen L., Marshall J.L. (2020). Disease-associated astrocytes in Alzheimer’s disease and aging. Nat. Neurosci..

[4] Zhang Y., Sloan S.A., Clarke L.E., Caneda C., Plaza C.A., Blumenthal P.D., Vogel H., Steinberg G.K., Edwards M.S., Li G. (2016). Purification and Characterization of Progenitor and Mature Human Astrocytes Reveals Transcriptional and Functional Differences with Mouse. Neuron.

[5] Coppe J.P., Desprez P.Y., Krtolica A., Campisi J. (2010). The senescence-associated secretory phenotype: The dark side of tumor suppression. Annu. Rev. Pathol..

[6] Bhat R., Crowe E.P., Bitto A., Moh M., Katsetos C.D., Garcia F.U., Johnson F.B., Trojanowski J.Q., Sell C., Torres C. (2012). Astrocyte senescence as a component of Alzheimer’s disease. PLoS ONE.

[7] Limbad C., Oron T.R., Alimirah F., Davalos A.R., Tracy T.E., Gan L., Desprez P.Y., Campisi J. (2020). Astrocyte senescence promotes glutamate toxicity in cortical neurons. PLoS ONE.

[8] Hayashide L.S., Pessoa B., Dias G., Pontes B., Pinto R.S., Diniz L.P. (2026). From Neuron-Centric to Glia-Centric: How Aging Glial Networks Drive Neurodegenerative Disease. J. Neurochem..

[9] Marques M., Hayashide L.S., Amorim P., Fernandes B.M., Araujo A.P.B., Messor D.F., Leocadio V.E., Pessoa B., Correa J., Villablanca C. (2025). Doxorubicin Induces a Senescent Phenotype in Murine and Human Astrocytes. J. Neurochem..

[10] Pickles S., Vigie P., Youle R.J. (2018). Mitophagy and Quality Control Mechanisms in Mitochondrial Maintenance. Curr. Biol. CB.

[11] Li W., Gui Y., Guo C., Huang Y., Liu Y., Yu X., Zhang H., Wang J., Liu R., Mahaman Y.A.R. (2025). Molecular mechanisms of mitochondrial quality control. Transl. Neurodegener..

[12] Diniz L.P., Araujo A.P.B., Carvalho C.F., Matias I., de Sa Hayashide L., Marques M., Pessoa B., Andrade C.B.V., Vargas G., Queiroz D.D. (2024). Accumulation of damaged mitochondria in aging astrocytes due to mitophagy dysfunction: Implications for susceptibility to mitochondrial stress. Biochim. Biophys. Acta Mol. Basis Dis..

[13] Araujo A.P.B., Vargas G., Hayashide L.S., Matias I., Andrade C.B.V., de Carvalho J.J., Gomes F.C.A., Diniz L.P. (2024). Aging promotes an increase in mitochondrial fragmentation in astrocytes. Front. Cell. Neurosci..

[14] Popov A., Brazhe N., Morozova K., Yashin K., Bychkov M., Nosova O., Sutyagina O., Brazhe A., Parshina E., Li L. (2023). Mitochondrial malfunction and atrophy of astrocytes in the aged human cerebral cortex. Nat. Commun..

[15] Wischhof L., Mathew A.J., Bonaguro L., Beyer M., Ehninger D., Nicotera P., Bano D. (2024). Mitochondrial complex I inhibition enhances astrocyte responsiveness to pro-inflammatory stimuli. Sci. Rep..

[16] Du F., Yu Q., Chen A., Chen D., Yan S.S. (2018). Astrocytes Attenuate Mitochondrial Dysfunctions in Human Dopaminergic Neurons Derived from iPSC. Stem Cell Rep..

[17] Hayakawa K., Esposito E., Wang X., Terasaki Y., Liu Y., Xing C., Ji X., Lo E.H. (2016). Transfer of mitochondria from astrocytes to neurons after stroke. Nature.

[18] Zhou J., Zhang L., Peng J., Zhang X., Zhang F., Wu Y., Huang A., Du F., Liao Y., He Y. (2024). Astrocytic LRP1 enables mitochondria transfer to neurons and mitigates brain ischemic stroke by suppressing ARF1 lactylation. Cell Metab..

[19] Dagda R.K., Cherra S.J., Kulich S.M., Tandon A., Park D., Chu C.T. (2009). Loss of PINK1 function promotes mitophagy through effects on oxidative stress and mitochondrial fission. J. Biol. Chem..

[20] Wiemerslage L., Lee D. (2016). Quantification of mitochondrial morphology in neurites of dopaminergic neurons using multiple parameters. J. Neurosci. Methods.

[21] Scholz D., Poltl D., Genewsky A., Weng M., Waldmann T., Schildknecht S., Leist M. (2011). Rapid, complete and large-scale generation of post-mitotic neurons from the human LUHMES cell line. J. Neurochem..

[22] Vazquez-Villasenor I., Garwood C.J., Simpson J.E., Heath P.R., Mortiboys H., Wharton S.B. (2021). Persistent DNA damage alters the neuronal transcriptome suggesting cell cycle dysregulation and altered mitochondrial function. Eur. J. Neurosci..

[23] Taguchi N., Ishihara N., Jofuku A., Oka T., Mihara K. (2007). Mitotic phosphorylation of dynamin-related GTPase Drp1 participates in mitochondrial fission. J. Biol. Chem..

[24] English K., Shepherd A., Uzor N.E., Trinh R., Kavelaars A., Heijnen C.J. (2020). Astrocytes rescue neuronal health after cisplatin treatment through mitochondrial transfer. Acta Neuropathol. Commun..

[25] Park J.H., Nakamura Y., Li W., Hamanaka G., Arai K., Lo E.H., Hayakawa K. (2021). Effects of O-GlcNAcylation on functional mitochondrial transfer from astrocytes. J. Cereb. Blood Flow Metab. Off. J. Int. Soc. Cereb. Blood Flow Metab..

[26] Belanger M., Allaman I., Magistretti P.J. (2011). Brain energy metabolism: Focus on astrocyte-neuron metabolic cooperation. Cell Metab..

[27] Buckman J.F., Hernandez H., Kress G.J., Votyakova T.V., Pal S., Reynolds I.J. (2001). MitoTracker labeling in primary neuronal and astrocytic cultures: Influence of mitochondrial membrane potential and oxidants. J. Neurosci. Methods.

[28] Sultana P., Honc O., Hodny Z., Novotny J. (2025). Clusterin Deficiency Promotes Cellular Senescence in Human Astrocytes. Mol. Neurobiol..

[29] Kim D., Yoo S.H., Yeon G.B., Oh S.S., Shin W.H., Kang H.C., Lee C.K., Kim H.W., Kim D.S. (2024). Senescent Astrocytes Derived from Human Pluripotent Stem Cells Reveal Age-Related Changes and Implications for Neurodegeneration. Aging Dis..

[30] Singh M.K., Shin Y., Han S., Ha J., Tiwari P.K., Kim S.S., Kang I. (2024). Molecular Chaperonin HSP60: Current Understanding and Future Prospects. Int. J. Mol. Sci..

[31] Zhu W., Cheng Y., Lang Z., Li W., Wei X. (2025). Astrocytic HSP60 Deletion Induced Astrocyte Senescence and Inhibited Neuroregeneration via Modulating the S1P/Truncated-BDNF Pathway. J. Neurosci. Res..

[32] Pellerin L., Magistretti P.J. (1994). Glutamate uptake into astrocytes stimulates aerobic glycolysis: A mechanism coupling neuronal activity to glucose utilization. Proc. Natl. Acad. Sci. USA.

[33] Magistretti P.J., Allaman I. (2015). A cellular perspective on brain energy metabolism and functional imaging. Neuron.

[34] Voloboueva L.A., Suh S.W., Swanson R.A., Giffard R.G. (2007). Inhibition of mitochondrial function in astrocytes: Implications for neuroprotection. J. Neurochem..

[35] Simmnacher K., Krach F., Schneider Y., Alecu J.E., Mautner L., Klein P., Roybon L., Prots I., Xiang W., Winner B. (2020). Unique signatures of stress-induced senescent human astrocytes. Exp. Neurol..

[36] Morales-Rosales S.L., Santin-Marquez R., Posadas-Rodriguez P., Rincon-Heredia R., Montiel T., Librado-Osorio R., Luna-Lopez A., Rivero-Segura N.A., Torres C., Cano-Martinez A. (2021). Senescence in Primary Rat Astrocytes Induces Loss of the Mitochondrial Membrane Potential and Alters Mitochondrial Dynamics in Cortical Neurons. Front. Aging Neurosci..

[37] Gong Q.Y., Wang W., Cai L., Jing Y., Yang D.X., Yuan F., Tian H.L., Ding J., Chen H., Xu Z.M. (2024). Transplantation of astrocyte-derived mitochondria into injured astrocytes has a protective effect following stretch injury. Mitochondrion.

[38] Joshi A.U., Minhas P.S., Liddelow S.A., Haileselassie B., Andreasson K.I., Dorn G.W., Mochly-Rosen D. (2019). Fragmented mitochondria released from microglia trigger A1 astrocytic response and propagate inflammatory neurodegeneration. Nat. Neurosci..

[39] Zhang C., Liu C., Li F., Zheng M., Liu Y., Li L., Yang H., Zhang S., Wang C., Rong H. (2022). Extracellular Mitochondria Activate Microglia and Contribute to Neuroinflammation in Traumatic Brain Injury. Neurotox. Res..

