# Supplementary material for: Astrocyte Senescence Disrupts the Extracellular Mitochondrial Compartment and Compromises Bioenergetic Support to Human Neurons
Source: Antioxidants (Basel). 2026 Sep 6;15(9):1127. doi: 10.3390/antiox15091127 (PMC13603318; doi:10.3390/antiox15091127)
Supplement: Supplementary file 1 [file antioxidants-15-01127-s001.zip › antioxidants-4505379-supplementary.pdf]

SUPPLEMENTARY FIGURES

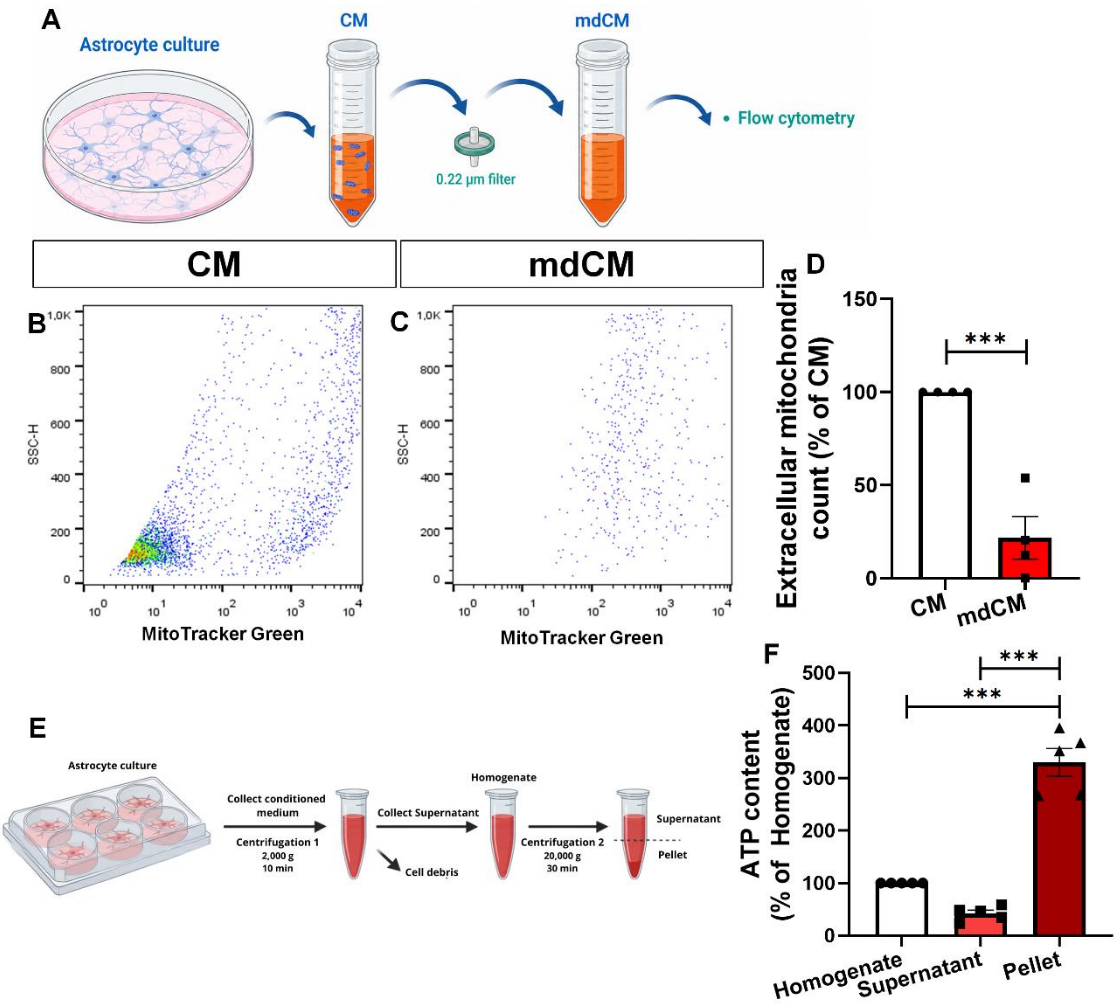

Supplementary Figure 1. Filtration and differential centrifugation characterize extracellular mitochondrial-associated material in human astrocyte-conditioned medium. (A) Schematic representation of the generation of mitochondria-depleted conditioned medium. Conditioned medium (CM) collected from primary human astrocytes was passed through a sterile 0.22-µm syringe filter to generate mitochondria-depleted conditioned medium (mdCM). Samples were incubated with MitoTracker Green and analyzed by flow cytometry. (B, C) Representative flow-cytometry plots showing side scatter (SSC-H) and MitoTracker Green fluorescence in unfiltered CM and mdCM. (D) Quantification of MitoTracker Green-positive extracellular particles, expressed relative to unfiltered CM. (E) Schematic representation of the differential centrifugation protocol used to fractionate astrocyte-conditioned medium. Following removal of cellular debris at 2,000 × *g* for 10 min, the resulting supernatant was centrifuged at 20,000 × *g* for 30 min to obtain a particulate pellet and the corresponding supernatant. (F) ATP content measured in the indicated conditioned-medium fractions. Data are presented as mean ± SEM. In (D), statistical significance was determined using a *t*-test comparing CM and mdCM. In (F), statistical significance was assessed by one-way ANOVA followed by Bonferroni's multiple-comparisons test, with pairwise comparisons performed between homogenate and supernatant, homogenate and pellet, and supernatant and pellet. ***$p < 0.001$.

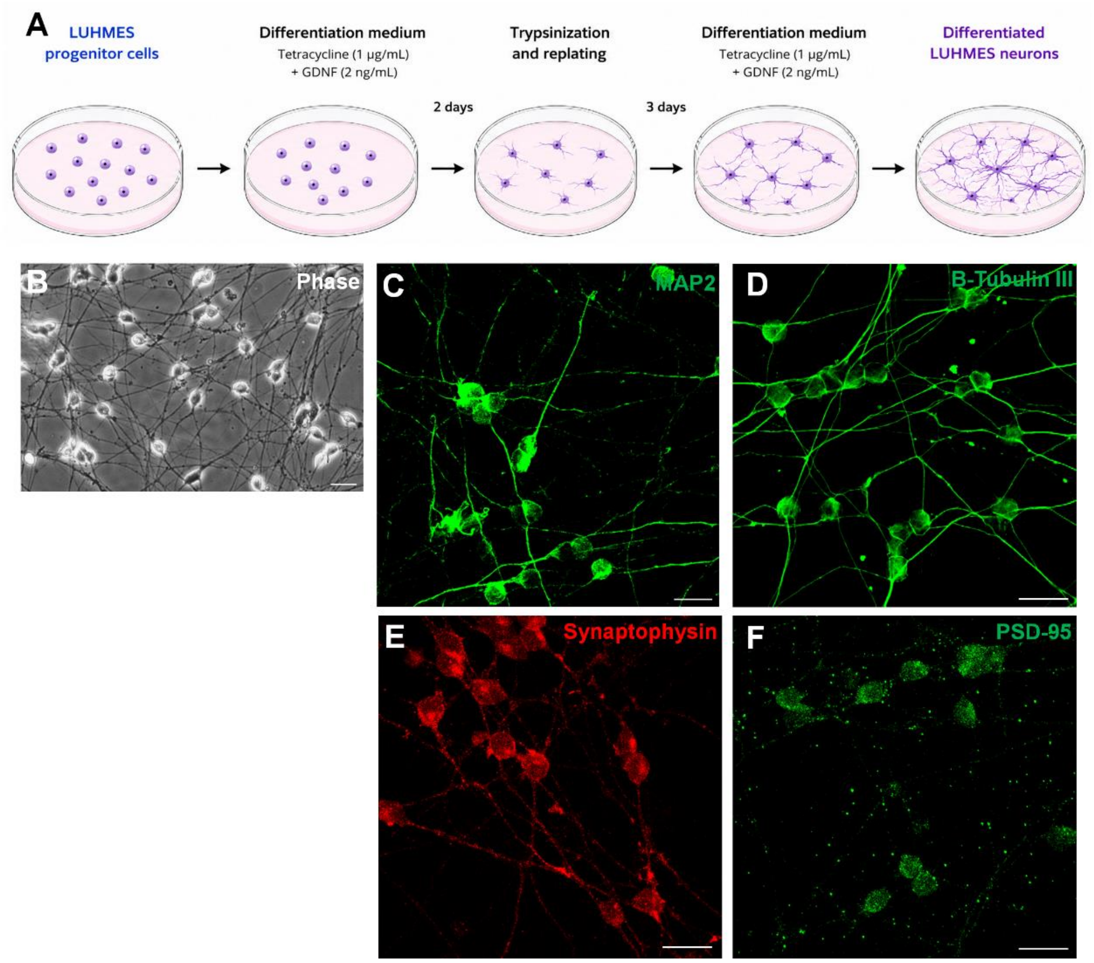

Supplementary Figure 2. Differentiation and phenotypic characterization of human LUHMES-derived neurons. (A) Schematic representation of the LUHMES differentiation protocol. Proliferating LUHMES progenitor cells were maintained in differentiation medium containing tetracycline (1 µg/mL) and glial cell line-derived neurotrophic factor (GDNF, 2 ng/mL) for 2 days. Cells were then trypsinized, replated and maintained in differentiation medium for an additional 3 days, resulting in a total differentiation period of 5 days. (B) Representative phase-contrast image showing the morphology and extensive neuritic network of differentiated LUHMES cells. (C–F) Representative fluorescence images showing expression of microtubule-associated protein 2 (MAP2, C), βIII-tubulin (D), synaptophysin (E) and postsynaptic density protein 95 (PSD-95, F). Scale bars, 20 µm.

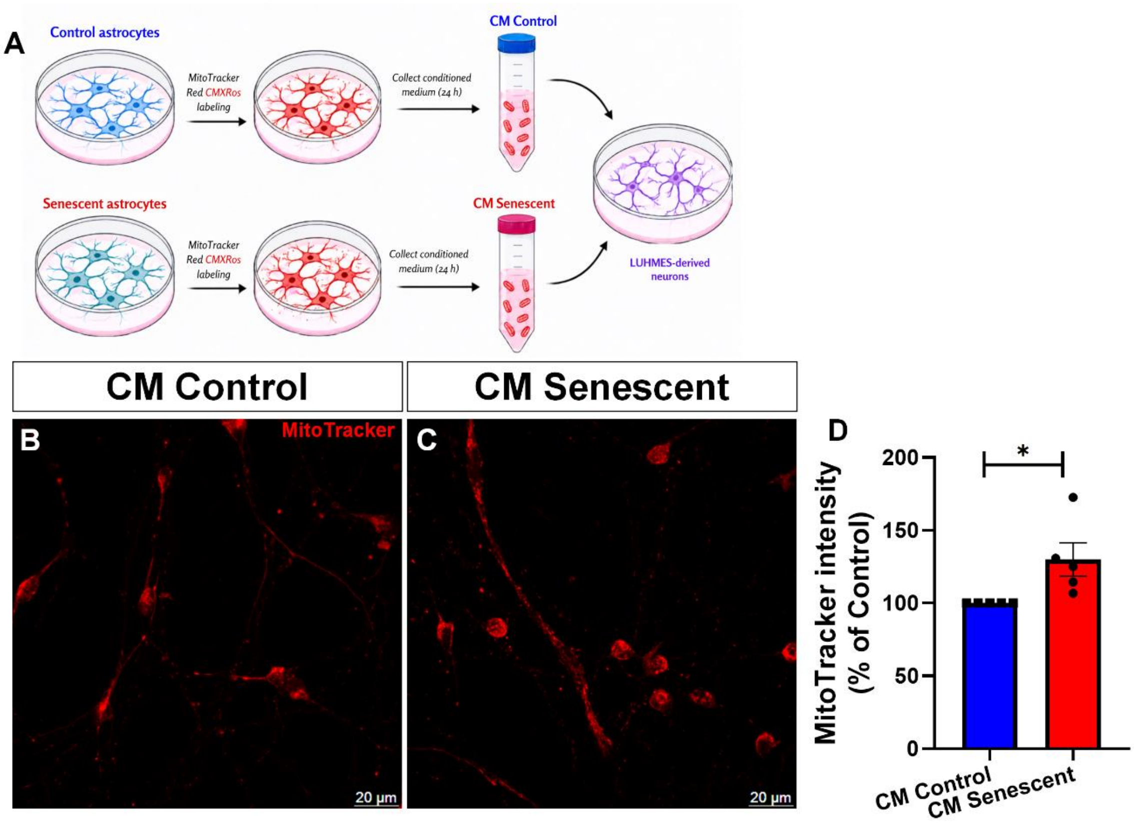

Supplementary Figure 3. Human LUHMES-derived neurons acquire MitoTracker-labeled mitochondrial material released by human astrocytes. (A) Schematic representation of the experimental protocol. Control and doxorubicin-induced senescent astrocytes were labeled with MitoTracker Red CMXRos for 1 h, thoroughly washed, and maintained in fresh medium for 24 h. Conditioned medium from control astrocytes (CM Control) or senescent astrocytes (CM Senescent) was subsequently collected and applied to differentiated LUHMES-derived neurons for 24 h. (B, C) Representative fluorescence images showing astrocyte-derived MitoTracker signal in neurons exposed to CM Control or CM Senescent. (D) Quantification of MitoTracker fluorescence intensity in recipient neurons, expressed relative to the CM Control group. Data are mean ± SEM from five independent cultures per group. Statistical significance was determined using *t*-test. *p < 0.05. Scale bars, 20 µm.
